# Mass Spectrometry Imaging of Kidney Tissue Sections of Rat Subjected to Unilateral Ureteral Obstruction

**DOI:** 10.1038/srep41954

**Published:** 2017-02-03

**Authors:** Huihui Liu, Wan Li, Qing He, Jinjuan Xue, Jiyun Wang, Caiqiao Xiong, Xiaoping Pu, Zongxiu Nie

**Affiliations:** 1 Key Laboratory of Analytical Chemistry for Living Biosystems, Institute of Chemistry Chinese Academy of Sciences, Beijing 100190, China; 2 Department of Molecular and Cellular Pharmacology, School of Pharmaceutical Sciences, Peking University, Beijing 100091, China; 3 University of Chinese Academy of Sciences, Beijing 100049, China; 4 Beijing Center for Mass Spectrometry, Beijing 100190, China

## Abstract

Chronic kidney disease (CKD) poses a serious threat to the quality of human life and health with an increasing incidence worldwide. Renal fibrosis is closely related to CKD and regarded as the final common pathophysiological pathway in most cases of end-stage renal diseases. Elucidating the mechanisms underlying renal fibrosis and developing novel therapeutic strategies are of great importance. Herein, matrix assisted laser desorption/ionization mass spectrometry imaging (MALDI MSI) based on 1, 5-diaminonaphthalene hydrochloride was applied to the rat model of unilateral ureteral obstruction (UUO) to investigate metabolic changes during renal fibrosis. Among identified endogenous compounds, twenty-one metabolites involved in metabolic networks such as glycolysis, tricarboxylic acid (TCA) cycle, ATP metabolism, fatty acids metabolism, antioxidants, and metal ions underwent relatively obvious changes after 1 and 3 weeks of UUO. Unique distribution of the metabolites was obtained, and metabolic changes of kidneys during renal fibrosis were investigated simultaneously for the first time. These findings once again highlighted the promising potential of the organic salt matrix for application in small molecule *in situ* MSI and in the field of biomedical research.

The kidneys play a critical role in keeping the body healthy not only by filtering the blood and getting rid of waste products, but also are involved in balancing the electrolyte levels and having the function of endocrine. The incidence of chronic kidney disease (CKD) is escalating worldwide and it has become a public health problem of global concern. Regardless of the initial pathogenesis of CKD, renal fibrosis is the inevitable common pathophysiological alteration in every progressive CKD, and it shows almost identical manifestation in all progressive forms of CKD with high clinical prevalence. unilateral ureteral obstruction (UUO) is a well-established experimental rodent model which can provided many new insights into the pathogenesis of obstructive nephropathy, and of progressive renal fibrosis in general^1^.

Considerable efforts have been devoted to studying the pathogenesis of renal fibrosis. The pathophysiology of renal fibrosis is described as follows^2^: (1) cellular activation and injury phase or priming; (2) fibrogenic signaling phase or activation; (3) fibrogenic phase or execution, with accumulation of extracellular matrix (ECM); and (4) destructive phase or progression. A growing body of evidence by biological technologies has indicated that the progression of renal fibrosis involves various molecular signaling pathways, such as TGF-β/Smad^3^,^4^, Wnt/β-catenin^5^, p38MAPK^6^, extracellular signal regulated kinase 1/2^6^, and cyclic nucleotide (cyclic adenosine monophosphate (cAMP) and cyclic guanosine monophosphate (cGMP))^7^. These previous studies facilitated us to better understand the process of renal fibrosis at the macrobiomolecular level. Furthermore,subtle changes in expression levels of these genes and proteins will no doubt be amplified and embodied in small molecule metabolites. Thus, analytical tools such as nuclear magnetic resonance (NMR) spectroscopy and mass spectrometry can be used for investigation of metabolomics in renal fibrosis. More recently, some researchers using ^1^H-NMR and UPLC-Q-TOF MS found alterations in the levels of some small molecule metabolites during renal fibrosis^8^,^9^,^10^,^11^.

Although these recent advances have led to a much better understanding of this disease, the pathophysiological mechanisms of renal fibrosis are extremely complicated and our ability to prevent and cure this disease remains limited up to now. Furthermore, altered metabolic pathways and networks during the progress of renal fibrosis especially regarding the spatial localization which is indispensable for accurately understanding complex pathogenesis cannot be obtained by using conventional analytical and biological techniques. Therefore, in-depth understanding of the molecular mechanisms of renal fibrosis is most essential and important, not only for elucidate the mechanism underlying renal fibrosis, but also for exploring and validating the efficacious therapies in order to reduce its morbidity and mortality worldwide.

Matrix assisted laser desorption/ionization mass spectrometry imaging (MALDI MSI), introduced by Caprioli in 1997^12^, has emerged as one of the most powerful technologies among the numerous techniques in recent years due to its unique features, namely, no necessity of labeling (free of label), high sensitivity, high throughout, molecule-specific, and the ability of *in situ* localizing a wide range of biomolecules simultaneously from a tissue specimen in one single run. Accompanied with technological and methodological improvements, MALDI-MSI has been more and more extensively used in the field of biomedical research^13^,^14^,^15^,^16^.

It is generally accepted that MALDI analysis is to a large extent dependent on the choice of matrix. Organic salts as novel matrix developed by our group in recent years possessed brilliant features including low cost, strong ultraviolet absorption, high salt tolerance ability, and fewer background signals especially in the low mass range (m/z < 500), which has potential application in small molecule MALDI analysis and *in situ* MSI^14^,^17^,^18^,^19^,^20^. 1, 5-diaminonaphthalene (1, 5-DAN) hydrochloride assisted LDI MSI of small molecules in brain tissue of rats subjected to middle cerebral artery occlusion (MCAO) previously carried out by our group successfully investigated the altered metabolic pathways and mechanisms underlying the development of ischemic brain damage^20^. In order to obtain as much information simultaneously about altered metabolic pathways and networks underlying renal fibrosis, a rat model of UUO was established in the present study and 1, 5-DAN hydrochloride assisted LDI MSI of small molecules in kidney tissues was carried out to visualize the spatial distribution and alteration of small molecule metabolites.

## Results

### Biochemical Analysis

**Figure 1:**
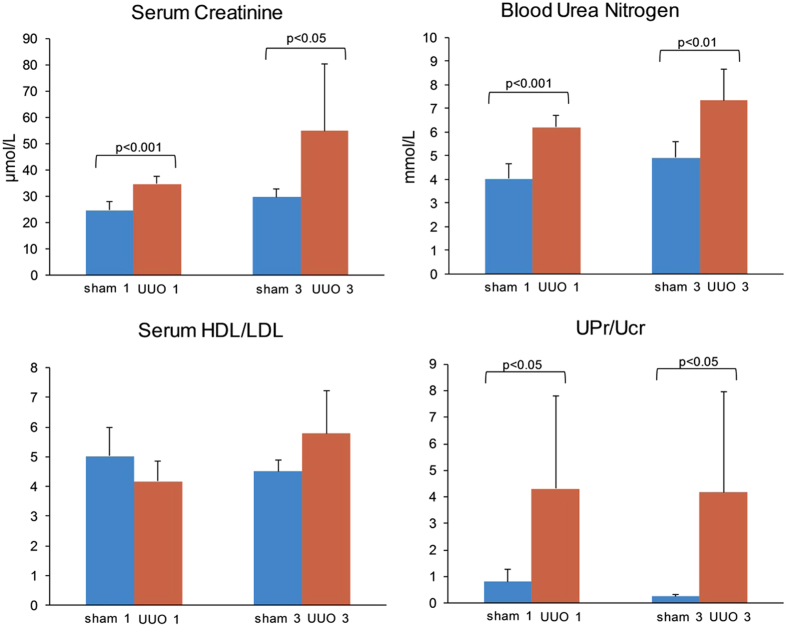
The biochemical indicators including serum creatinine, blood urea nitrogen, HDL/LDL and UPr/Ucr ratio in sham-operated and UUO rats. The data represent the mean ± SD. UPr: urine protein; Ucr: urine creatinine; HDL: high-density lipoprotein; LDL: low-density lipoprotein; Sham 1: sham-operation 1-week group; Sham 3: sham-operation 3-week group; UUO 1: UUO 1-week group; UUO 3: UUO 3-week group. n = 5.

Concentrations of the urine protein and creatinine, serum creatinine, blood urea nitrogen (BUN), and serum high-density lipoprotein (HDL) and low-density lipoprotein (LDL) detected using biochemical assay kits were shown in Fig. 1 and Table S1. The results demonstrated that there was a significant difference in contents of serum creatinine, BUN, and urine protein and urine creatinine ratio (UPr/Ucr) between the sham-operation groups and UUO groups. The serum creatinine concentrations were 1.41-fold (P < 0.001) and 1.85-fold (P < 0.05) increased in UUO 1-week and 3-week group, respectively, compared with corresponding control group. Levels of BUN were 1.54-fold (P < 0.001) and 1.49-fold (P < 0.01) higher in UUO 1-week and 3-week group, respectively, compared with corresponding control group. The UPr/Ucr increased 5.25-fold (P < 0.05) and 15.71-fold (P < 0.05) in UUO 1-week and 3-week group, respectively, compared with corresponding sham-operated group. Serum HDL and LDL have not significant difference between sham-operated group and UUO group (P > 0.05).

### Histopathological Changes in the Kidneys of UUO Rats

**Figure 2:**
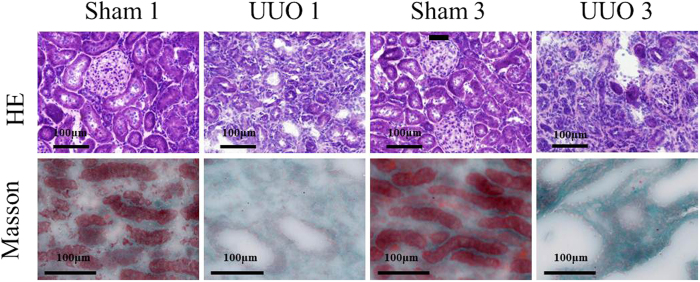
HE and Masson’s trichrome staining of Sprague-Dawley rat kidneys at 1 and 3 weeks after UUO (n = 5).

The hematoxylin eosin (HE) and Masson’s trichrome stainings of kidneys obtained from sham-operated rats and UUO rats are shown in Fig. 2 and Figure S1. As shown in Figure S1, the kidneys have been swelled 1-week post UUO and the kidneys tissues were not intact with morphology being destroyed compared with the control group. The situation worsened with the obstruction progression to 3-week. As HE staining demonstrated, the structure of rat kidneys tissues was clear and intact in the control group, whereas the UUO group displayed glomerular atrophy. Furthermore, the number of stroma cells increased and inflammatory cells infiltrated in the interstitium accompanied with fibrosis in the UUO group. In the UUO 3-week group, the pathological changes were much more obvious and local bubble like deformation appeared. Masson’s trichrome staining revealed massive cell necrosis and dissolution in the UUO group. Furthermore, the intact renal morphology and structure has been destroyed and replaced by fibrous tissue in the UUO group. The situation was much more severe in the UUO 3-week group.

### MSI of Rat Kidneys Following Fibrosis

**Figure 3:**
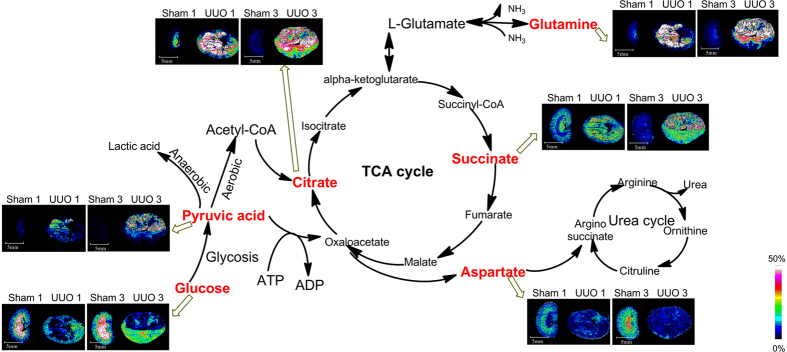
*In situ* MALDI MSI distribution and changes of metabolites involved in glycosis and TCA cycle in the kidneys from sham-operated group and UUO group. Rat kidneys were removed and fresh frozen under −80 °C. Tissues were sectioned at 10 μm thickness and then used for *in situ* metabolite imaging. Mass imaging data were acquired in negative ionization mode with spatial resolution of 200 μm. All imaging data were normalized with the total ion chromatogram. Scale bar: 5 mm.

**Figure 4:**
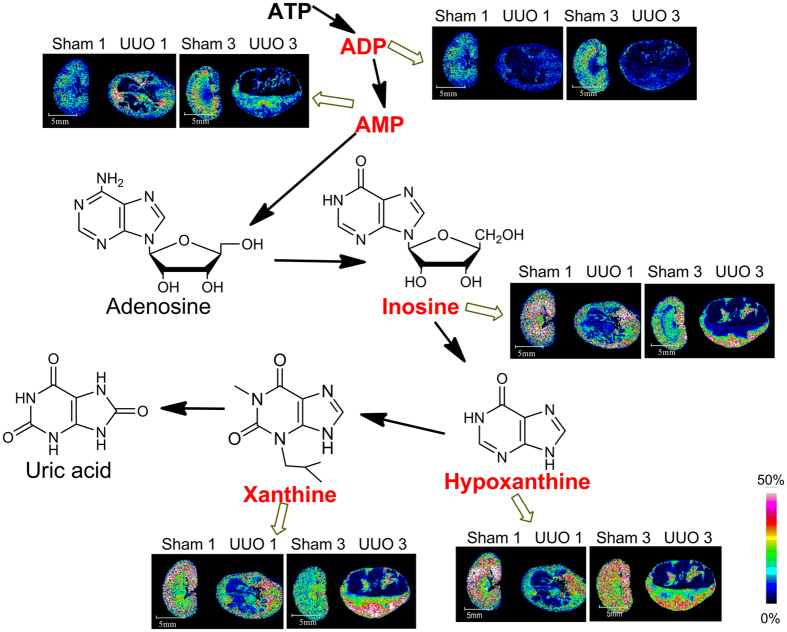
*In situ* MALDI MSI distribution and changes of metabolites involved in ATP metabolism. Scale bar: 5 mm.

**Figure 5:**
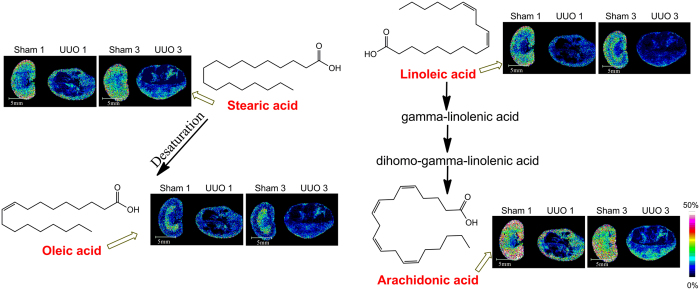
*In situ* MALDI MSI distribution and changes of fatty acids in the kidneys from control group and UUO group. Scale bar: 5 mm.

**Figure 6:**
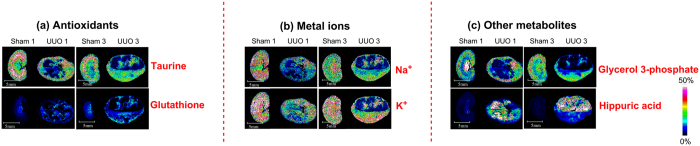
*In situ* MALDI MSI distribution and changes of (**a**) antioxidants, (**b**) metal ions, and (**c**) other identified metabolites in the kidneys from control group and UUO group. Scale bar: 5 mm.

Represented mass spectra of kidney sections from control group and UUO group using 1, 5-DAN hydrochloride as matrix by MALDI TOF MS were shown in Figure S2. Accurate mass by MALDI Fourier transform ion cyclotron resonance (FTICR) were summarized in Table S2. MS/MS data of metabolites were presented in Table S3. Among the identified small molecules, metabolites involved in glycolysis, tricarboxylic acid (TCA) cycle, ATP metabolism, fatty acids metabolism, and antioxidants as well as metal ions underwent relatively significant changes in the UUO group compared with the sham-operated group. MSI maps of relative changes of these metabolites were shown in Figure S3 and Figs 3–6.

#### Perturbation on Glycolysis

Glucose is a very important substance involved in the glycolysis. The unique distribution and changes of glucose shown in Fig. 3 and Figure S3a demonstrated that the concentration of glucose in renal medulla is far higher than that in renal cortex in the sham-operation group. The present study suggested glucose level in renal medulla especially in the damaged area decreased remarkably after 1 week and 3 weeks of UUO compared with the sham-operation group. The changes of glucose concentration might be due to altered transport or utilition. The concentration of glucose is known to affect the expression of ECM protein^21^ which is very important to renal fibrosis. Pyruvic acid is the end product of the anaerobic portion of glycolysis. As demonstrated in Fig. 3 and Figure S3b, the concentration of pyruvic acid relatively increased after 1 week and 3 weeks of UUO and the metabolite mainly accumulated in the destroyed area. Statistics based on all tested samples shown in Figure S3a and b confirmed these findings.

#### Interruption of TCA Cycle

Citric acid is a very important intermediate in the TCA cycle. As shown in Fig. 3 and Figure S3c, the concentration of citric acid in renal pelvis is relatively higher than that in renal cortex and renal medulla in the sham-operation group. Furthermore, levels of citric acid was observed to increase obviously in the kidney section especially in the destroyed area after 1 week and 3 weeks of UUO compared with the sham-operation group. Succinate, another well-known intermediate in the TCA cycle, is converted by succinate dehydrogenase to generate fumarate. The succinate dehydrogenase complex is part of the electron transport chain in the mitochondrial membrane. Mitochondrial dysfunction in case of renal fibrosis might ultimately lead to changes of succinate. As shown in Fig. 3 and Figure S3d, relative amount of succinate showed relative increase 1 week after surgery, compared with the sham-operation group. Though levels of succinate in four individuals subjected to relative increase after 3 weeks of UUO, statistics showed no significance. Glutamine is metabolite of α-ketoglutaric acid which is an important intermediate in the TCA cycle. The concentration of glutamine in renal medulla is relatively higher than that in renal cortex in the sham-operation group. After 1 week and 3 weeks of UUO, glutamine was observed to increase obviously especially in the destroyed area compared with the sham-operation group (Fig. 3 and Figure S3e). Aspartate is a common intermediate of TCA cycle and urea cycle. As shown in Fig. 3 and Figure S3f, level of aspartate remarkably decreased in the whole kidney section after 1 week and 3 weeks of UUO.

#### Disruption of ATP Metabolism

The spatial distribution and changes of metabolites involved in ATP metabolism in kidney sections of rats subjected to UUO for 1 week and 3 weeks visualized by MSI are presented in Fig. 4 and Figure S3g–k. As shown in Fig. 4 and Figure S3g, the level of ADP showed relatively remarkable decrease after 1 week and 3 weeks of UUO. The amount of AMP in renal pelvis is relatively higher than that in renal parenchyma in the sham-operation group and the concentration of AMP relatively decreased especially in the destroyed area 3 weeks post-surgery (Seen in Fig. 4 and Figure S3h). Levels of inosine and hypoxanthine presented relative decrease especially in the destroyed area after 1 week and 3 weeks of UUO compared with the sham-operation group (Fig. 4 and Figure S3i and j). As presented in Figure S3k, among five rats subjected to UUO for 1 week, three individuals showed relative decreased levels for xanthine especially in the destroyed area whereas one of the left two displayed relative increased levels in the undamaged area and the other showed decreased level only in the destroyed area compared with the control group. However, the situation changed 3 weeks post UUO. Three individuals among five rats showed relative increased levels in undamaged area and relative decreased levels in damaged area. As to the left two rats, one displayed relative decreased levels only in the damaged area and the other showed relative increase in the undamaged area.

#### Disturbance of Fatty Acids Metabolism

As shown in Fig. 5 and Figure S3n–q, levels of four fatty acids including linoleic acid (LA), oleic acid, stearic acid and arachidonic acid (AA) also underwent significant changes during renal fibrosis. As shown in Fig. 5, Figure S3l and o, level of LA and AA in renal pelvis is relatively lower than that in renal cortex and renal medulla in the sham-operation group. Compared with the control group, concentrations of LA and AA showed relative decrease especially in the destroyed area after 1 week and 3 weeks of UUO. As shown in Fig. 5 and Figure S3m, level of oleic acid in renal medulla is relatively higher than that in renal cortex and renal pelvis in the control group. Compared with the sham-operation group, concentrations of oleic acid and stearic acid displayed relative decrease especially in the destroyed area after 1 week and 3 weeks of UUO (Figure S3m and n). The results indicated disturbance of fatty acid metabolism.

#### Effects on Antioxidants

MSI maps and alterations of low molecular weight antioxidants were obtained as shown in Fig. 6a. As shown in Figure S3p, the concentration of taurine in renal cortex is far higher than that in renal medulla. Compared with the sham-operation group, taurine showed relative decreased level especially in the destroyed area after 1 week of UUO and the situation has not been improved even 3 weeks post UUO. The typical distribution of GSH shown in FigureS2q demonstrated that the concentration of GSH in renal medulla is far higher than that in renal cortex in the sham-operation group. Compared with the sham-operation group, levels of GSH showed relative decrease in the destroyed area after 1-week and 3-week of UUO.

#### Disturbances of Ion Homeostasis

Rat kidneys subjected to UUO showed disturbances of ion homeostasis. As demonstrated in Fig. 6b and Figure S3r and s, among five rats subjected to UUO for 1 week, three individuals showed relative decreased levels for Na^+^ and K^+^ especially in the destroyed area whereas one of the left two displayed relative increased levels in the undestroyed area and the other showed no obvious change compared with the control group. After 3 weeks of UUO, relative decreased levels for Na^+^ and K^+^ in the destroyed area were observed.

#### Other Metabolic Changes

Except for altered metabolic changes mentioned above, two other identified metabolites, glycerol 3-phosphate and hippuric acid also showed changes during renal fibrosis (Fig. 6c and Figure S3t and u). Glycerol 3-phosphate is synthesized by reducing dihydroxyacetone phosphate (DHAP), a glycolysis intermediate, with glycerol-3-phosphate dehydrogenase. Compared with the sham-operation group, glycerol 3-phosphate displayed relative decrease especially in the destroyed area 1 week and 3 weeks post UUO (Fig. 6c and Figure S3t). Level of hippuric acid displayed relative increase and it mainly accumulated in the destroyed area after 1 week and 3 weeks of UUO compared with the sham-operation group (Fig. 6c and Figure S3u). Hippuric acid has been recognized as a potential marker of uremic toxicity in chronic renal failure^22^.

## Discussion

Creatinine, generated from muscle metabolism, was filtered through the kidneys’ glomeruli and disposed in the urine. Thus, the creatinine level in blood and urine is a reliable indicator of kidney function. In case of renal fibrosis, renal inherent cells are damaged, leading to impaired function of glomerular filtration and renal tubular reabsorption, sequentially the elevated creatinine level in blood and decreased creatinine level in urine. Furthermore, as the function of glomerular filtration and renal tubular reabsorption damaged during the process of renal fibrosis, the urine proteins levels increased, resulting in elevated UPr/Ucr. BUN is a waste product of protein catabolism in the urea cycle and is removed from the blood by the kidneys. BUN level is another indicator of kidney function, which can build up if kidney function is impaired. Level changes of creatinine, UPr/Ucr, and BUN in UUO group displayed in Fig. 1 indicated impaired function of kidneys. All of the results together with the changes in the renal tissue by HE and Masson’s trichrome stainings suggested that the established UUO model was successful.

Kidney sections from rats in sham-operation group and UUO group were then subjected to MALDI MSI. Among the identified small molecules, metabolites involved in energy metabolism, fatty acids metabolism, and antioxidants as well as metal ions underwent relatively significant changes in the UUO group compared with the sham-operated group.

Malfunctioning mitochondria have received increasing attention as it is linked to numerous disorders^23^. Mitochondria are the energy factories of the cells, serving to sustain energy. The mitochondrial energy metabolism involves three essential sets of biochemical reactions: the respiratory chain (RC), the TCA cycle, and the ATP synthesis machinery. The end product of the glycolysis was converted to acetyl coenzyme A under aerobic conditions to enter the TCA cycle. TCA cycle, a central part of the energetic metabolism that contributes to the generation of ATP, involves a series of enzyme-catalyzed chemical reactions. ATP mainly generated through oxidative phosphorylation in the mitochondria and was catabolized to be ADP, AMP, adenosine, inosine, hypoxanthine, xanthine, uric acid following two catabolic routes known as the “IMP pathway” and the “adenosine pathway”^24^. The mitochondria distributed in cells vary greatly, depending on the distinct energy demands of different tissues. The kidneys is rich in mitochondria and highly demands of energy. Therefore, mitochondrial dysfunction of the kidney plays a critical role in the pathogenesis of kidney diseases^25^ and it is an early event that occurs prior to the onset of renal fibrosis^26^. MSI maps and alterations of metabolites involved in glycolysis, TCA cycle, and ATP metabolism indicated interruption of energy metabolism and mitochondrial dysfunction of the kidney.

Except for energy-related metabolites, the present study showed relative changes for LA, AA, oleic acid and stearic acid after 1 week and 3 weeks of UUO (Fig. 5). Fatty acids are important sources of fuel because, they are broken down to CO_2_ and water by the intra-cellular mitochondria, releasing large amounts of energy, captured in the form of ATP through beta oxidation and the TCA cycle. As we all know, LA is known to be an essential fatty acid which cannot be synthesized by human or can only be synthesized very little. The present study observed distribution of LA in kidneys, which were rarely reported previously. Saline perfusions were carried out to fully wash off the blood before kidneys dissection in order to avoid the interference from the metabolites of bloodstream. So the possibility of metabolites in kidney pick up from bloodstream is very little. Wang *et al*. previously reported distribution of LA in mouse brain and liver^14^. Furthermore, LA was screened as one of the biomarkers related to renal fibrosis by Li *et al*.^27^. These previous studies provided strong support for our results. AA, a polyunsaturated fatty acid (PUFA) present in the membrane phospholipids, can be biosynthesized from LA. In addition to being involved in cellular signaling as a lipid second messenger for regulation of signaling enzymes, AA can also act as a key inflammatory intermediate. Furthermore, PUFAs have been reported to be associated with the development of chronic kidney disease and PUFA supplementation can reduce renal inflammation and fibrosis in animal models^28^. Oleic acid is a fatty acid that occurs naturally in various animal and vegetable fats and oils. Stearic acid is one of the useful types of saturated fatty acids that come from many animal and vegetable fats and oils. Conversion of stearic acid to oleic acid has been shown to take place in refed rats^29^. Alterations of these metabolites indicated interruption of fatty acids metabolism.

Figure 6a showed MSI maps and changes of antioxidants during renal fibrosis. Reactive oxidative species (ROS) has been proved to play a critical role in the pathogenesis of chronic renal disease such as diabetic nephropathy, proliferative glomerulo-nephritis, and renal fibrosis induced by transient ischemic injury, renal ablation, and ureteral obstruction^30^,^31^. Mitochondria are the primary intracellular source of ROS and dysfunction of the mitochondria leads to an overproduction of ROS, which sequentially results in kidney fibrosis by stimulating cellular transformation to myofibroblasts^32^. Low molecular weight antioxidants, such as taurine, ascorbic acid and glutathione (GSH), are important endogenous mechanism as a protective response to the existing oxidative stress. It has been reported that taurine can suppressed the increase of infiltrating macrophages^33^ or act as an antioxidant^34^, leading to the attenuation of renal interstitial fibrosis.

It has been well-established that one important function of the kidneys is balancing the electrolyte levels, and thus impaired function of rat kidneys subjected to UUO might lead to disturbances of ion homeostasis. Figure 6c showed MSI maps and alterations of Na^+^ and K^+^ after 1 week and 3 weeks of UUO. One of the mechanisms was suggested to be the dependency of tubular sodium reabsorption on the activity of Na^+^/K^+^ ATPase, which plays a crucial role in regulating renal sodium handling and maintaining ion homeostasis. ATP is a fundamental energy source for sustaining the function of Na^+^/K^+^ ATPase. Dysfunctional mitochondria in case of renal fibrosis led to depletion of ATP and eventually suppression of Na^+^/K^+^ ATPase^35^. Furthermore, it has been reported that renal tubular expression of Na/K-ATPase rapidly decreased in kidneys after UUO, contributing to compromised sodium regulation^36^.

In summary, UUO model was established and 1, 5-DAN hydrochloride assisted LDI MSI of small molecules in kidney tissue sections permitted us to simultaneously visualize unique distributions and alterations of metabolites during renal fibrosis. The results demonstrated interruption of glycosis, TCA cycle, ATP metabolism, fatty acids metabolism, antioxidants, and ion homeostasis were observed in the process of renal fibrosis. The newly prepared organic salt matrix possesses unique features, which promises its potentially effective and widespread application in small molecule *in situ* MSI and in the field of biomedical research.

## Methods

### Chemicals and Reagents

1, 5-DAN for matrix preparation, and pelltobarbitalum natricum for anesthesia were purchased from Sigma-Aldrich (St. Louis, MO). Standards including lactic acid, pyruvic acid, succinate, aspartate, taurine, glutamine, hypoxanthine, xanthine, ascorbic acid, citric acid, creatinine, glucose, glutathione, inosine, AMP, ADP, ATP, were also purchased from Sigma-Aldrich (St. Louis, MO).

### Animals

Twenty male healthy Sprague-Dawley rats (200 ± 10 g) were purchased from the Institute of Laboratory Animal Science, Chinese Academy of Medical Science & Peking Union Medical College. All animals were maintained in an environmentally controlled room under controlled temperature (20–24 °C) and relative humidity (40–70%) with a 12-h light/dark cycle. Standard diet and water were provided to the rats ad libitum. The animal experiments were performed according to the NIH Guide for the Care and Use of Laboratory Animals (National Institutes of Health Publication, No. 3040–2, revised 1999, Bethesda, MD) and were approved by the Animal Care and Use Committee of the Chinese Academy of Sciences.

### Establishment of the UUO Model

After acclimatization of one week prior to experiments, the rats were randomly divided into four groups with five rats for each group: UUO group (model group, 1-week and 3-week) and sham-operated group (control group, 1-week and 3-week). Rats were anaesthetized by abdominal injection of 2% pelltobarbitalum natricum (40 mg/kg by body weight). After routine sterilization and shaving, a longitudinal incision was made on the left side of the abdomen and the left kidney was exposed. The left ureter was isolated and the end close to the renal pelvis was permanently ligatured twice with 4–0 silk suture. The middle of this ligatured part was cut. Then the organs were relocated and the incision was sutured. The sham-operated control group rats underwent the same surgical operation except for ureter ligation.

### Sample Collection

Samples were collected at 1 week and 3 weeks after the operation (n = 5 rat per group). Urine was collected from the residuary ureter linked to the kidney for 2.5 hours before the rats were sacrificed and immediately centrifuged at 5000 g for 10 min. The supernatant was separated and stored at −80 °C until analysis. Just before the rats were sacrificed, 2–3 mL of blood samples were collected via angular vein and centrifuged at 3000 g for 20 min at 4 °C to obtain serum. In order to avoid the interference from the metabolites of bloodstream, saline perfusions were carried out to fully wash off the blood before kidneys dissection. The left kidney was then removed and snap-frozen in liquid nitrogen. All tissues were stored at −80 °C until further preparation.

### Biochemical Analysis

Analysis of urine and serum were performed at the Deyi Diagnostics Corporation (Beijing, China). Concentrations of the urine protein and creatinine, serum creatinine, blood urea nitrogen, serum high-density lipoprotein (HDL) and low-density lipoprotein (LDL) were measured. Then, after 24 hours, the urine protein-to-creatinine ratio (Up/Ucr) was calculated.

### Histopathology Staining

For histopathology, the harvested kidneys of all rats were sectioned at 10 μm thickness using a Leica CM1950 cryostat (Leica Microsystems GmbH, Wetzlar, Germany) at −18 °C and were stained with HE to reveal histopathological lesions. Kidney fibrosis was also evaluated by Masson’s trichrome staining.

### Mass Spectrometry Imaging

The harvested kidneys were fixed atop a drop of saline on the cutting stage. Tissues were sectioned at 10 μm thickness using a Leica CM1950 cryostat (Leica Microsystems GmbH, Wetzlar, Germany) at −18 °C and thaw mounted onto indium tin oxide (ITO) coated glass slides. The glass slides were then placed into a vacuum desiccator for approximately 1 h before matrix application.

For MSI, the matrix solution, 1, 5-DAN hydrochloride in 50% ethanol/water prepared as literature reported^20^, was sprayed onto the tissue sections mounted onto ITO coated glass slides using an automatic matrix sprayer (ImagePrep, Bruker Daltonics) and made sure to have homogeneous matrix coverage over the entire tissue surface.

MALDI MSI experiments were performed on an Ultraflextreme MALDI-TOF/TOF MS (Bruker Daltonics, Billerica, MA) equipped with a smartbeam Nd: YAG 355 nm laser operating at 2000 kHz. Medium focus was set for the laser spot size (laser spot diameter of ~50 μm), and laser power was optimized prior to each run and then fixed during the whole experiment. The mass spectra were acquired in negative reflectron mode with a pulsed ion extraction time of 130 ns, an accelerating voltage of 20.00 kV, an extraction voltage of 17.55 kV, a lens voltage of 6.4 kV, and a reflector voltage of 21.10 kV. The mass spectra data were acquired in the mass range from m/z 50 to 1000 Da. The imaging data for each array position consists of 200 laser shots with spatial resolution being 200 μm for all kidney tissue sections. External mass calibration was performed with standards before data acquisition. MALDI mass spectra were normalized with the total ion current (TIC), and the signal intensity of each imaging data displayed was the normalized intensity. MS/MS fragmentations performed on the Ultraflextreme MALDI-TOF/TOF MS in the LIFT^TM^ mode together with FTICR MS were used for further detailed structural confirmation of the identified metabolites^20^.

### Statistical Analysis

Regions of interest were defined and corresponding average intensity of metabolites were acquired. Two-tailed Student’s t test was performed to compare average intensity of metabolites between UUO and sham control group or different regions of kidney tissue. P-values ≤ 0.05 were considered statistically significant.

## Additional Information

**How to cite this article**: Liu, H. *et al*. Mass Spectrometry Imaging of Kidney Tissue Sections of Rat Subjected to Unilateral Ureteral Obstruction. *Sci. Rep.* **7**, 41954; doi: 10.1038/srep41954 (2017).

**Publisher's note:** Springer Nature remains neutral with regard to jurisdictional claims in published maps and institutional affiliations.

## Supplementary Material

Supplementary Information
