# Supplementary material for: Mass Spectrometry Imaging of Kidney Tissue Sections of Rat Subjected to Unilateral Ureteral Obstruction
Source: Sci Rep. 2017 Feb 3;7:41954. doi: 10.1038/srep41954 (PMC5291210; doi:10.1038/srep41954)
Supplement: Supplementary Information [file srep41954-s1.pdf]

Supplementary Information

Mass Spectrometry Imaging of Kidney Tissue Sections of Rat Subjected to Unilateral Ureteral Obstruction

Huihui Liu[1], Wan Li[2], Qing He[1,3], Jinjuan Xue[1,3], Jiyun Wang[1], Caiqiao Xiong[1], Xiaoping Pu[2] & Zongxiu Nie[1,3,4,*]

[1]Key Laboratory of Analytical Chemistry for Living Biosystems, Institute of Chemistry Chinese Academy of Sciences, Beijing 100190, China

[2]Department of Molecular and Cellular Pharmacology, School of Pharmaceutical Sciences, Peking University, Beijing 100191, China

[3]University of Chinese Academy of Sciences, Beijing 100049, China

[4]Beijing Center for Mass Spectrometry, Beijing 100190, China

* Corresponding author: znie@iccas.ac.cn (Z. Nie).

Supplementary Information - Methods

HE staining. The kidneys tissue sections were subjected to HE staining in order to evaluate the histopathological lesions of the UUO model. The staining procedures are as follows:

1. Fixed in cold acetone (4℃) for 20 seconds.
2. Hydrate in running tap water for 1 minute.
3. Stain in Harris hematoxylin solution for 8 minutes.
4. Wash in running tap water for 5 minutes.
5. Differentiate in 1% acid ethanol for 30 seconds.
6. Wash in running tap water for 1 minute.
7. Bluing in 1% ammonia water for 30 seconds.
8. Wash in running tap water for 3 minutes, and distilled water for 1 minute.
9. Counterstain in eosin-phloxine solution for 20 seconds.
10. Wash in distilled water for 1 minute.
11. Dehydrate through 75% ethanol for 2 min, 85% ethanol for 2 min, 95% ethanol for 2 min, and 2 changes of absolute ethanol with 5 minutes each.
12. Clear in 2 changes of xylene, 5 minutes each.
13. Mount with resinous mounting medium.

Masson's trichrome staining. The kidneys tissue sections were also subjected to Masson's trichrome staining in order to evaluate the kidney fibrosis. The staining procedures are as follows:

1. Fixed in 75% ethanol (room temperature) for 10 minutes.

2. Hydrate in distilled water for 1 minute.

3. Stain in Ponceau S/ Acid Fuchsin solution for 5 minutes.

4. Wash in 0.2% acetic acid for 1 minute.

6. Differentiation in Phosphomolybdic/Phosphotungstic Acid Solution for 2 minutes.

7. Wash in 0.2% acetic acid for 1 minute.

8. Stain in Brilliant Green (1%) for 1 minute.

9. Wash in 0.2% acetic acid for 1 minute.

10. Dehydrate through 95% ethanol for 10 seconds, and 2 changes of absolute alcohol with 10 seconds each.

12. Clear in 2 changes of xylene, 5 minutes each.

13. Mount with resinous mounting medium.

MS/MS Spectra. On-tissue MS/MS fragmentation of metabolites acquired using the LIFT technique was carried out on the Ultraflextreme MALDI-TOF/TOF mass spectrometer. The laser operated at 1000 Hz and the MS/MS spectra were the sum of 5000 shots for precursor ions and 10000 shots for product ions, respectively. MS/MS spectra in the reflector mode were acquired with a pulsed ion extraction time of 80 ns, an accelerating voltage of 7.5 kV, an extraction voltage of 6.75 kV, a lens voltage of 3.5 kV, a reflector voltage of 29.50 kV, a lift 1 voltage of 19.00 kV and a lift 2 voltage of 4.2 kV.

MALDI FT-ICR MS. A Bruker 9.4 T solariX FT-ICR mass spectrometer equipped with an Apollo dual-mode electrospray ionization (ESI)/MALDI ion source, with a

355 nm and 200 Hz solid-state Smartbeam Nd:YAG UV laser (Bruker Daltonics, Bremen, Germany) was employed for recording accurate masses of metabolites in kidney sections from rats. Mass spectra were acquired over the mass range from 50 to 1000 Da in negative ion mode. For MALDI-MS profiling, the mass spectra were recorded by accumulating 20 shots at 200 laser shots per scan.

Supplementary Information – Tables and Figures

Table S1. The serum creatinine, blood urea nitrogen, HDL/LDL and UPr/Ucr ratio measured in sham-operated and UUO rats. n=5.

Group	Serum Creatinine (μmol/L)	BUN (mmol/L)	HDL/LDL	UPr/Ucr
	Mean ±SD	Mean ±SD	Mean ±SD	Mean ±SD
Sham 1	24.65 ±3.17	4.02 ±0.64	5.01 ±0.97	0.82 ±0.47
UUO 1	34.68 ±2.98***	6.19 ±0.49***	4.18 ±0.68	4.30 ±3.48*
Sham 3	29.63 ±2.91	4.90 ±0.68	4.51 ±0.39	0.26 ±0.05
UUO 3	54.83 ±25.25*	7.32 ±1.31**	5.79 ±1.44	4.16 ±3.81*

* $P < 0.05$, ** $P < 0.01$, *** $P < 0.001$, model group versus corresponding control group.

BUN: blood urea nitrogen;

HDL: high-density lipoprotein;

LDL: low-density lipoprotein;

UPr: urine protein;

Ucr: urine creatinine;

Sham 1: sham-operation 1-week group;

UUO 1: UUO 1-week group;

Sham 3: sham-operation 3-week group;

UUO 3: UUO 3-week group.

Table S2. Small molecular metabolites in kidneys identified by the accurate mass measurement using MALDI-FTICR MS in negative ion mode.

name	Parent ion	Theoretical m/z	Experimental m/z	delta	ppm
Taurine	$[M-H]^-$	124.007388	124.00729	-9.8E-05	-0.79
Aspartate	$[M-H]^-$	132.030232	132.03003	-0.000202	-1.53
Hypoxanthine	$[M-H]^-$	135.031234	135.03111	-0.000124	-0.92
Glutamine	$[M-H]^-$	145.061866	145.06188	1.4E-05	0.10
Glutamate	$[M-H]^-$	146.045882	146.04584	-4.2E-05	-0.29
Xanthine	$[M-H]^-$	151.026149	151.02621	6.1E-05	0.40
Glycerol 3-phosphate	$[M-H]^-$	171.006399	171.00643	3.1E-05	0.18
Ascorbic acid	$[M-H]^-$	175.024812	175.02477	-4.2E-05	-0.24
Hippuric acid	$[M-H]^-$	178.050967	178.0509	-6.7E-05	-0.38
Citric acid	$[M-H]^-$	191.019727	191.01968	-4.7E-05	-0.25
Glycerylphosphorylethanolamine/ sn-glycero-3-Phosphoethanolamine	$[M-H]^-$	214.048598	214.04859	-8E-06	-0.04
Glucose	$[M+Cl]^-$	215.032789	215.03297	0.000181	0.84
Inositol cyclic phosphate	$[M-H]^-$	241.011878	241.01191	3.2E-05	0.13
Inosine	$[M-H]^-$	267.073493	267.07343	-6.3E-05	-0.24
Linoleic acid	$[M-H]^-$	279.232954	279.23305	9.6E-05	0.34
Oleic acid	$[M-H]^-$	281.248604	281.24861	6E-05	0.07
Stearic acid	$[M-H]^-$	283.264254	283.26435	0.00015	0.17
Arachidonic acid	$[M-H]^-$	303.232954	303.23307	0.00017	0.19
Glutathione	$[M-H]^-$	306.07653	306.0766	7E-05	0.23
Galactosylhydroxylysine	$[M-H]^-$	323.14599	323.1462	0.00021	0.65
Docosahexaenoic acid	$[M-H]^-$	327.232954	327.23294	-1.4E-05	-0.04
Adenosine2’,3’-cyclic phosphate/ Cyclic AMP	$[M-H]^-$	328.045244	328.04542	0.000176	0.54
AMP	$[M-H]^-$	346.055808	346.05597	0.000162	0.47
Pantetheine 4'-phosphate	$[M-H]^-$	357.089082	357.08926	0.000178	0.50
GMP	$[M-H]^-$	362.050723	362.05076	3.7E-05	0.10
CPA(16:0)	$[M-H]^-$	391.225499	391.22578	0.000281	0.72
CPA(18:2)	$[M-H]^-$	415.225499	415.22572	0.000221	0.53
CPA(18:0)	$[M-H]^-$	419.2568	419.25699	0.00019	0.45
ADP	$[M-H]^-$	426.022139	426.02226	0.000121	0.28
LPA(18:0)	$[M-H]^-$	437.267364	437.26773	0.000366	0.84
LPE(16:0)	$[M-H]^-$	452.278263	452.27871	0.000447	0.99
LPE(18:0)	$[M-H]^-$	480.309563	480.31	0.000437	0.91
LPE(20:4)	$[M-H]^-$	500.278263	500.27833	6.7E-05	0.13
ATP	$[M-H]^-$	505.988469	505.98865	0.000181	0.36
LPI(18:0)	$[M-H]^-$	599.320188	599.32025	6.2E-05	0.10

name	Parent ion	Theoretical m/z	Experimental m/z	delta	ppm
Heme	$[M-H]^-$	615.170022	615.17014	0.000118	0.19
Hemin	$[M-H]^-$	650.138874	650.13886	-1.4E-05	-0.02
PA (16:0/18:1)	$[M-H]^-$	701.51268	701.51257	-0.00011	-0.16
PE (16:0/18:1)	$[M-H]^-$	716.52357	716.52306	-0.00051	-0.71

AMP: adenosine monophosphate, ADP: adenosine diphosphate, ATP: adenosine triphosphate, GMP: guanosine monophosphate, CPA: cyclic phosphatidic acid, LPA: lysophosphatidic acid, LPE: lysophosphatidylethanolamine, LPI: lysophosphatidylinositol, PA: phosphatidic acid, PE: phosphatidylethanolamine.

Table S3. MS/MS data of metabolites using MALDI-TOF/TOF MS in negative ion mode.

$[M-H]^-$	Structurally specific MS/MS peaks	Assignment
306	288, 270, 205, 130	Glutathione
346	327, 283, 211, 151, 133, 97	AMP
362	283, 211, 198, 97, 79	GMP
426	408, 346, 328, 283, 213, 176, 158, 133	ADP
506	426, 408, 327, 176, 158	ATP
452	255, 214, 196, 153, 140, 97	LPE(16:0)
480	420, 283, 255, 196, 153	LPE(18:0)
599	315, 283, 241, 153	PI(18:0)
650	571, 391, 283, 255, 123, 79	Hemin
701	419, 346, 281, 255, 153	PA (16:0/18:1)
716	452, 392, 281, 255, 168, 141	PE (16:0/18:1)

Figure S1. a) HE and b) Masson's trichrome staining of Sprague-Dawley rat kidneys at 1 and 3 weeks after UUO (n=5). Sham 1: sham-operation 1-week group; Sham 3: sham-operation 3-week group; UUO 1: UUO 1-week group; UUO 3: UUO 3-week group.

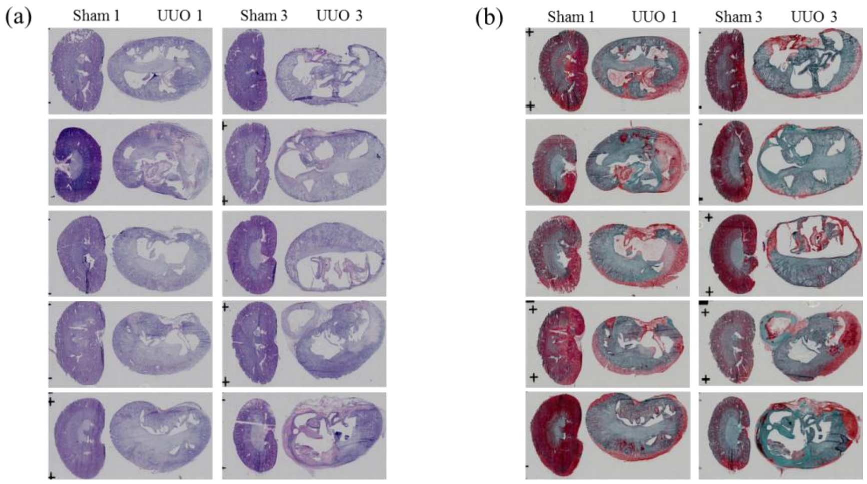

Figure S2. Represented MALDI mass spectra of kidney sections from control group and UUO group using 1, 5-DAN hydrochloride as matrix by MALDI TOF MS. (a) Matrix: 1, 5-DAN hydrochloride; (b) Sham 1: sham-operation 1-week group; (c) UUO 1: UUO 1-week group; (d) Sham 3: sham-operation 3-week group; (e) UUO 3: UUO 3-week group.

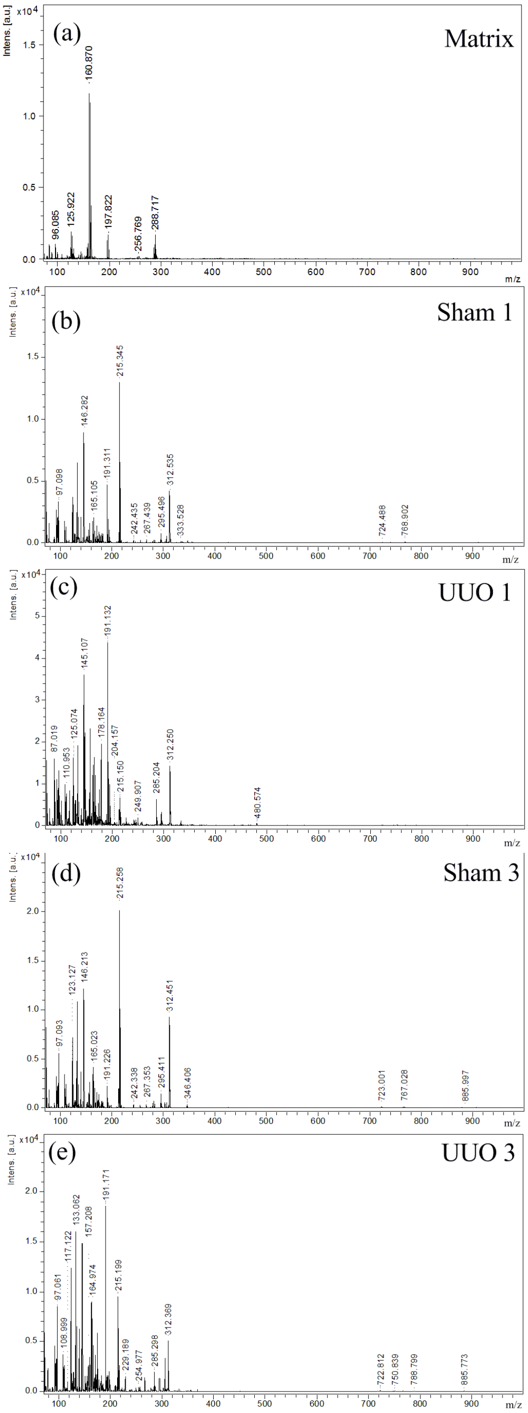

Figure S3. *In situ* MALDI MSI distribution and relative changes of metabolites in the kidney sections from control group and UUO group (n=5). Rat kidneys were removed and immediately frozen under - 80 ℃. One section at 10 μm thickness from rat subjected to UUO and one section from rats in control group were thraw-mounted onto one ITO-coated glass slide and then used for *in situ* metabolite imaging. Mass imaging data were acquired in negative ionization mode with 200 μm spatial resolution. All imaging data were normalized with the total ion chromatogram. Regions of interest were defined and corresponding average intensity was acquired. Two-tailed Student's t test was performed to compare average intensity of metabolites between UUO and sham control group or different regions of kidney tissue. P-values ≤0.05 were considered statistically significant. This figure consists of *in situ* MALDI MSI distribution of metabolites (upper MSI maps) and corresponding comparison of the average intensity between UUO and sham control group or different regions of kidney tissue (lower histograms). (a) $[M+Cl]^-$, m/z 215; (b) $[M-H]^-$, m/z 87; (c) $[M-H]^-$, m/z 191; (d) $[M-H]^-$, m/z 117; (e) $[M-H]^-$, m/z 145; (f) $[M-H]^-$, m/z 132; (g) $[M-H]^-$, m/z 426; (h) $[M-H]^-$, m/z 346; (i) $[M-H]^-$, m/z 267; (j) $[M-H]^-$, m/z 135; (k) $[M-H]^-$, m/z 151; (l) $[M-H]^-$, m/z 279; (m) $[M-H]^-$, m/z 281; (n) $[M-H]^-$, m/z 283; (o) $[M-H]^-$, m/z 303; (p) $[M-H]^-$, m/z 124; (q) $[M-H]^-$, m/z 306; (r) $[M+2Cl]^-$, m/z 93; (s) $[M+2Cl]^-$, m/z 109; (t) $[M-H]^-$, m/z 171; (u) $[M-H]^-$, m/z 178; Unfirmed-1: $[M-H]^-$, m/z 101; Unfirmed-2: $[M-H]^-$, m/z 148; Unfirmed-3: $[M+Cl]^-$, m/z 214; Unfirmed-4: $[M+Cl]^-$, m/z 246; Unfirmed-5: $[M+Cl]^-$, m/z 285. AMP: adenosine monophosphate, ADP: adenosine diphosphate, ATP: adenosine triphosphate. Sham 1: sham-operation 1-week group; Sham 3: sham-operation 3-week group; UUO 1: UUO 1-week group; UUO 3: UUO 3-week group. * $P < 0.05$, ** $P < 0.01$, *** $P < 0.001$. Scale bar: 5mm.

(a) Glucose

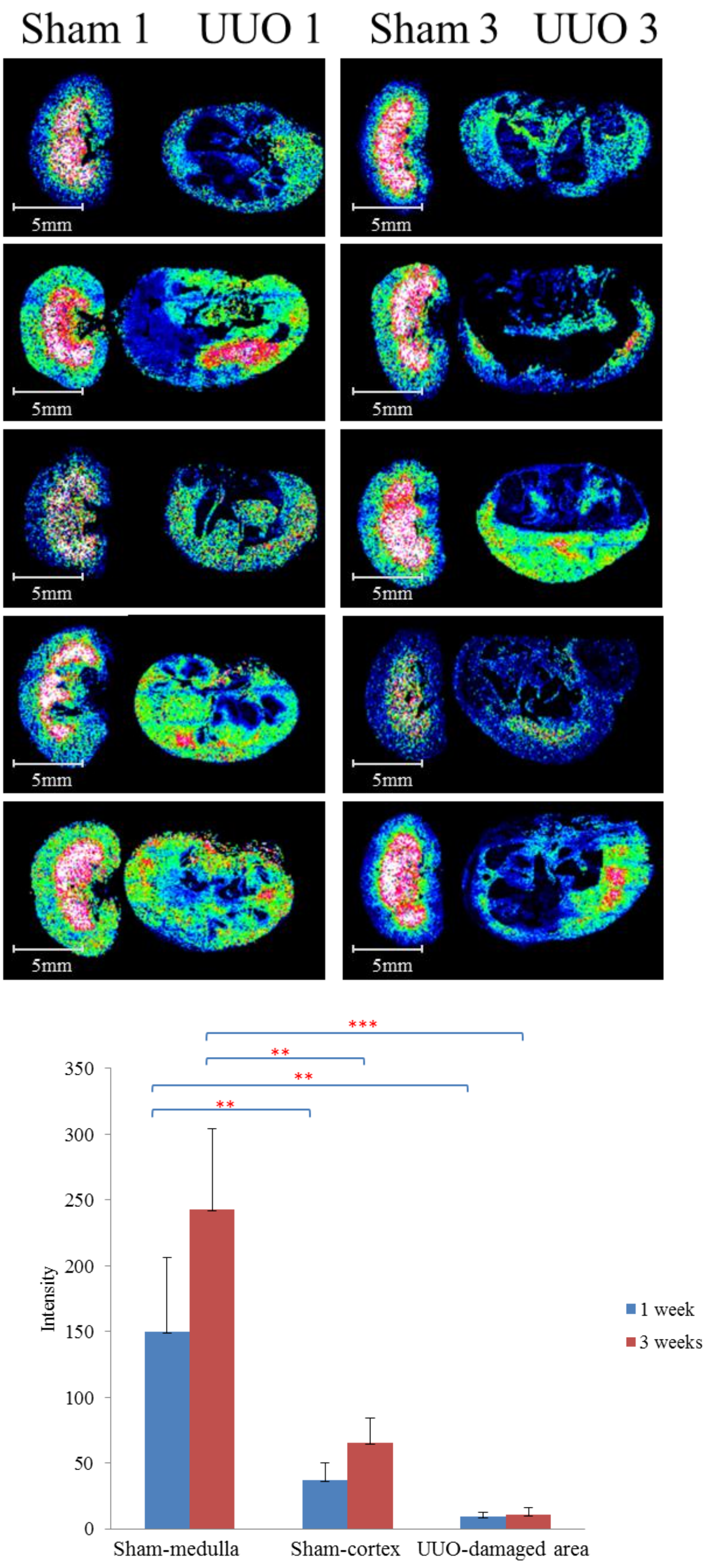
Sham 1
UUO 1
Sham 3
UUO 3
5mm
***
**
**
**
350
300
250
200
150
100
50
0
Intensity
1 week
3 weeks
Sham-medulla
Sham-cortex
UUO-damaged area

(b) Pyruvic acid

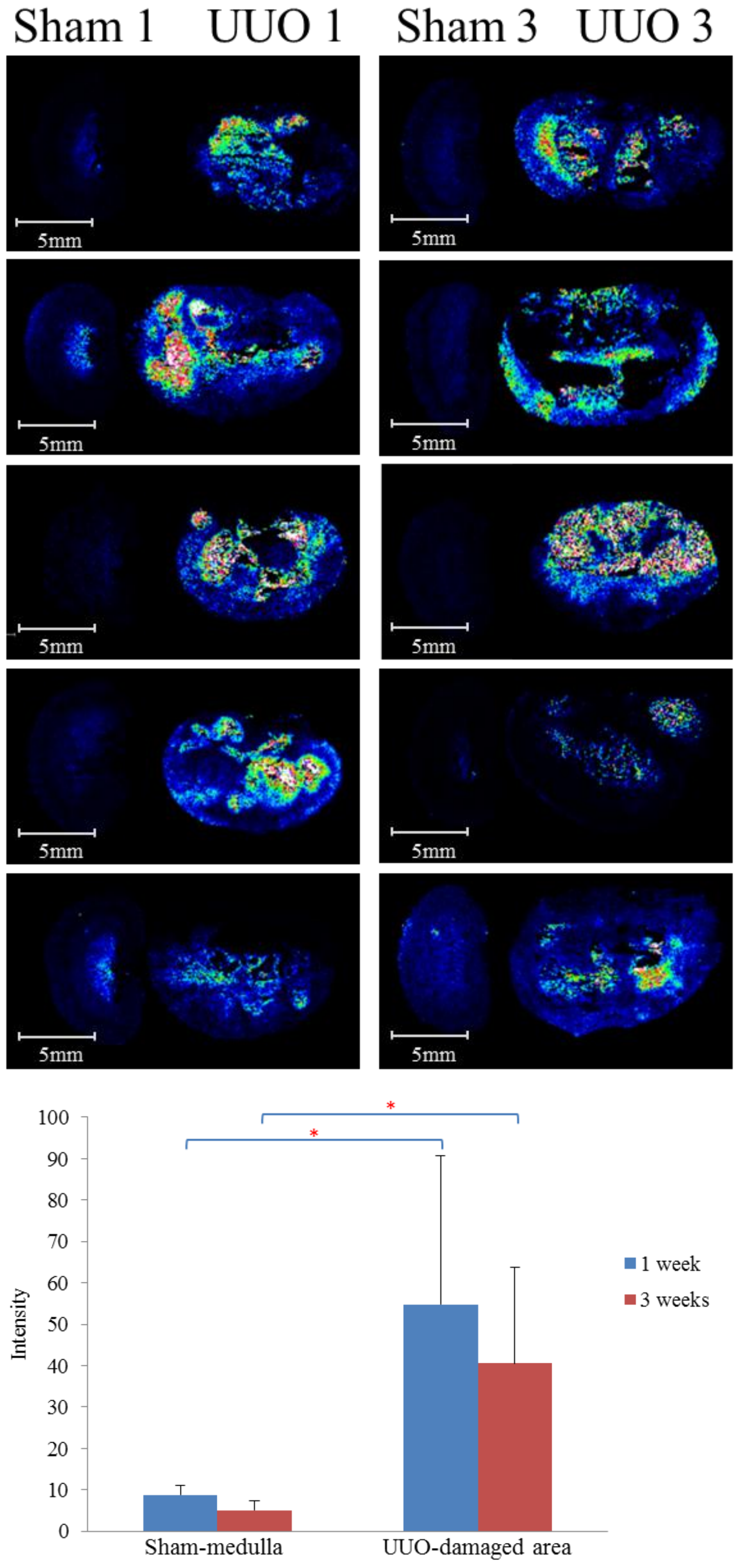

(c) Citric acid

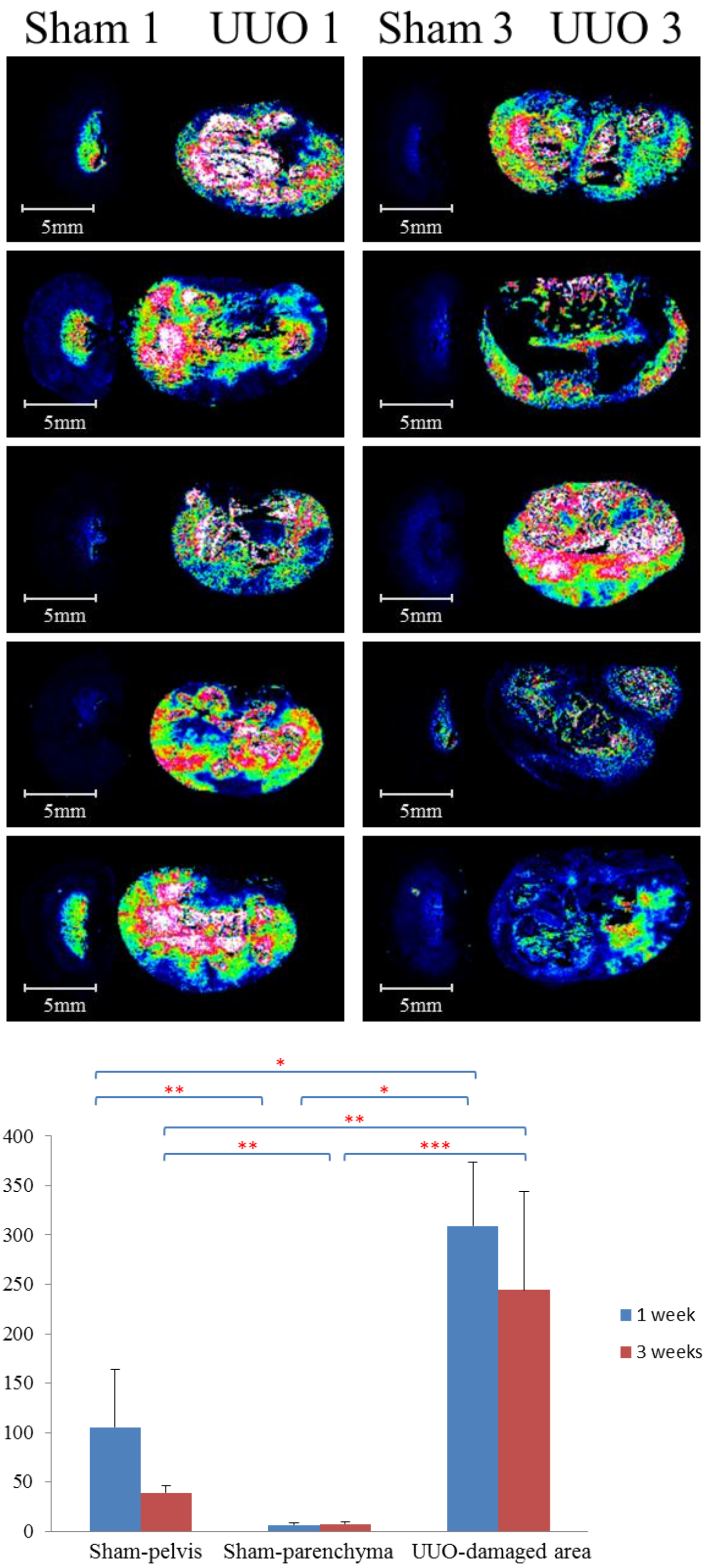

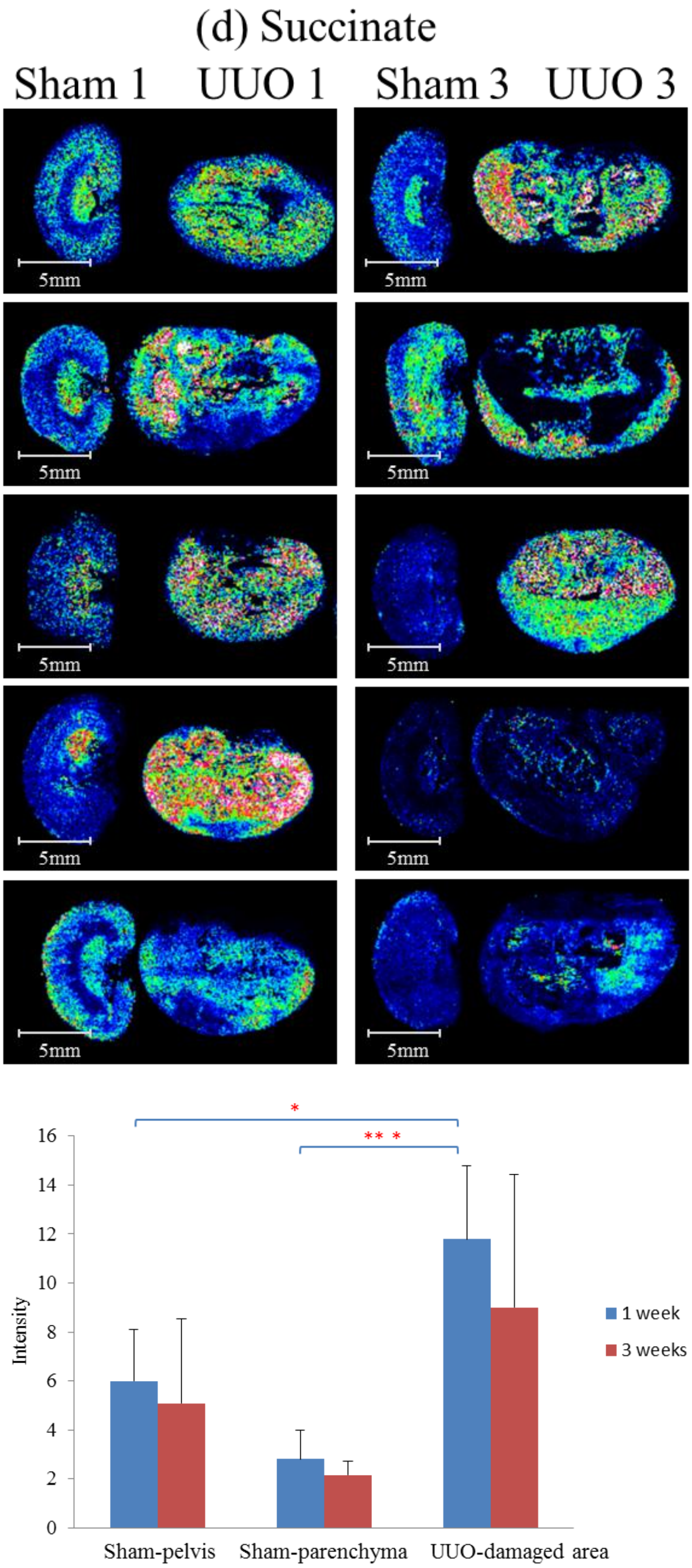
(d) Succinate
Sham 1
UUO 1
Sham 3
UUO 3
5mm
*
** *
Intensity
16
14
12
10
8
6
4
2
0
Sham-pelvis
Sham-parenchyma
UUO-damaged area
1 week
3 weeks

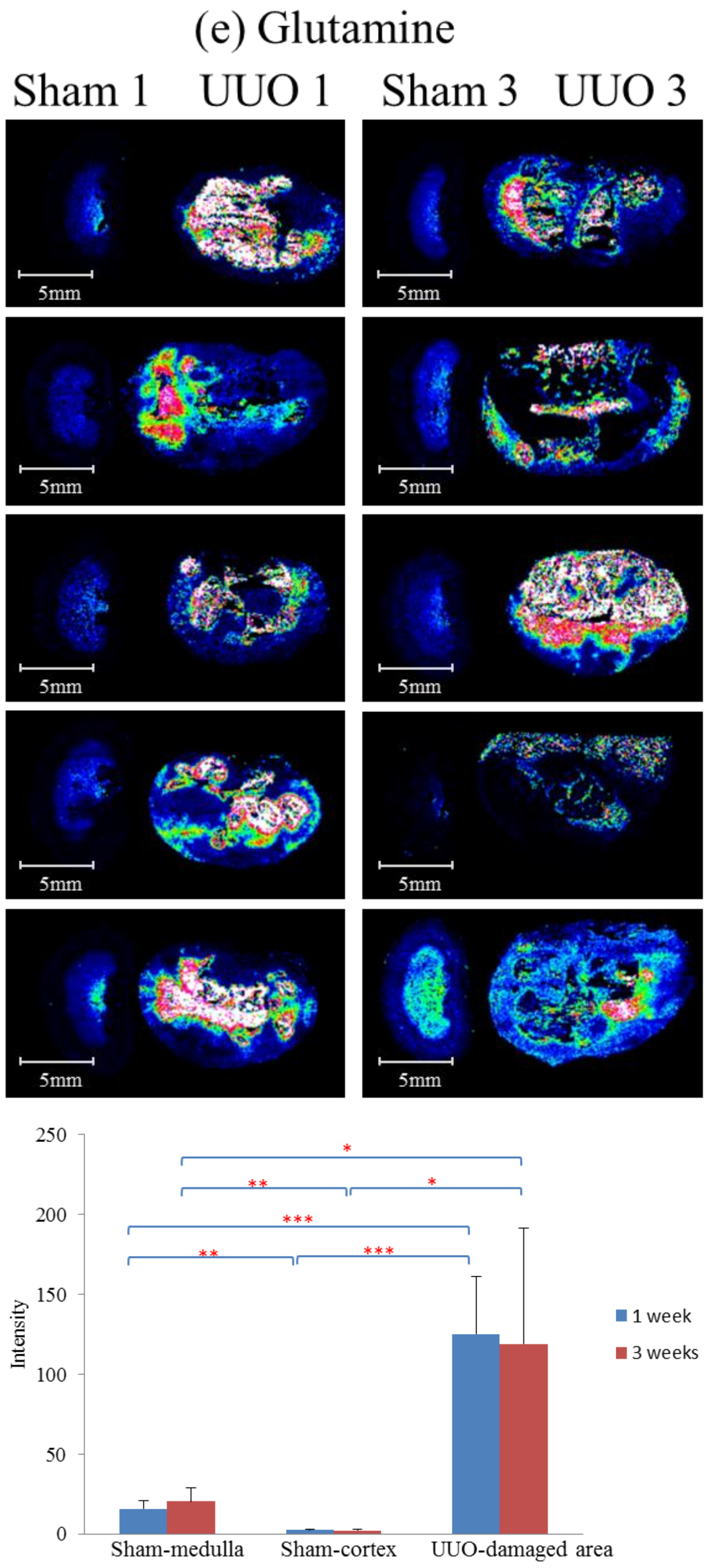
(e) Glutamine
Sham 1
UUO 1
Sham 3
UUO 3
5mm
250
200
150
100
50
0
Intensity
*
**
***
1 week
3 weeks
Sham-medulla
Sham-cortex
UUO-damaged area

(f) Aspartate

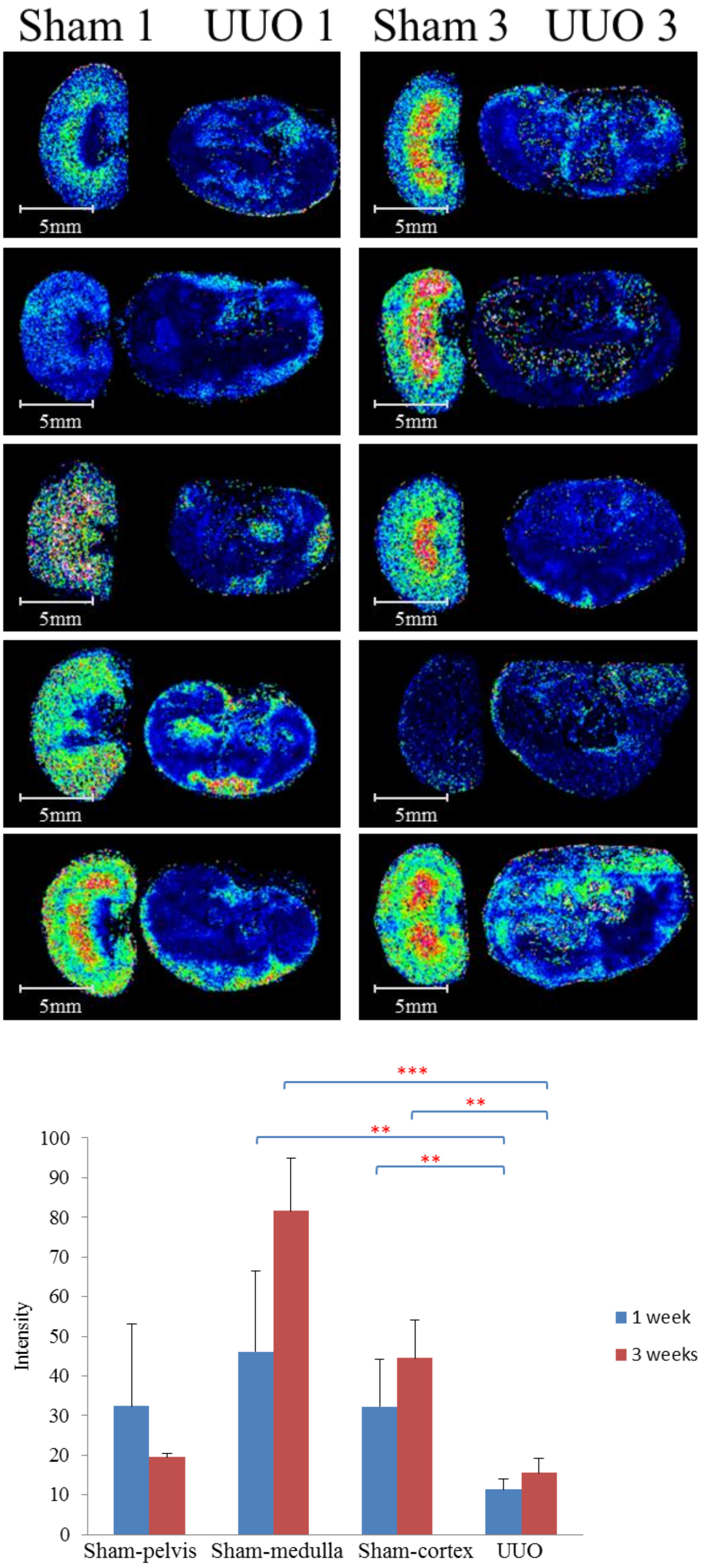
Sham 1
UUO 1
Sham 3
UUO 3
5mm
***
**
**
**
100
90
80
70
60
50
40
30
20
10
0
Intensity
Sham-pelvis
Sham-medulla
Sham-cortex
UUO
1 week
3 weeks

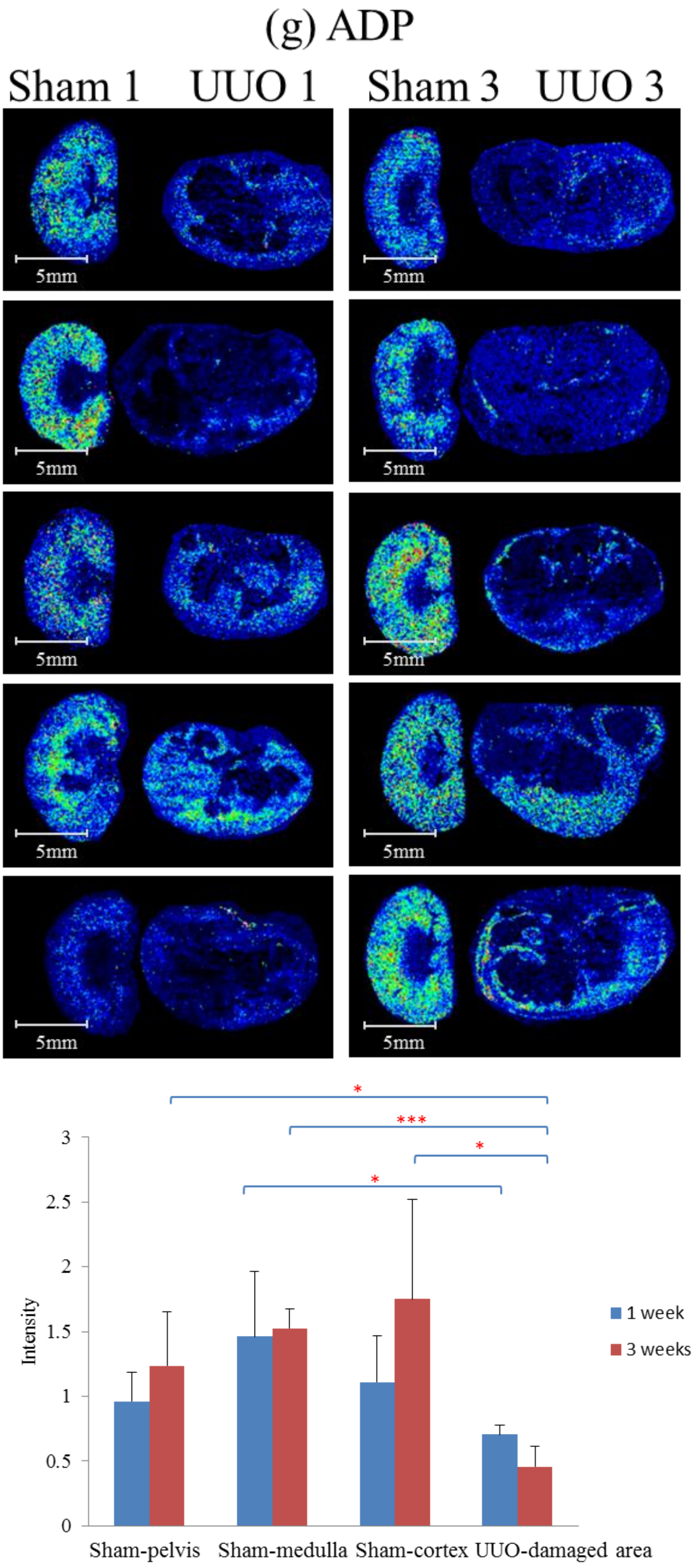
(g) ADP
Sham 1
UUO 1
Sham 3
UUO 3
5mm
*
***
*
*
Intensity
0
0.5
1
1.5
2
2.5
3
Sham-pelvis
Sham-medulla
Sham-cortex
UUO-damaged area
1 week
3 weeks

(h) AMP

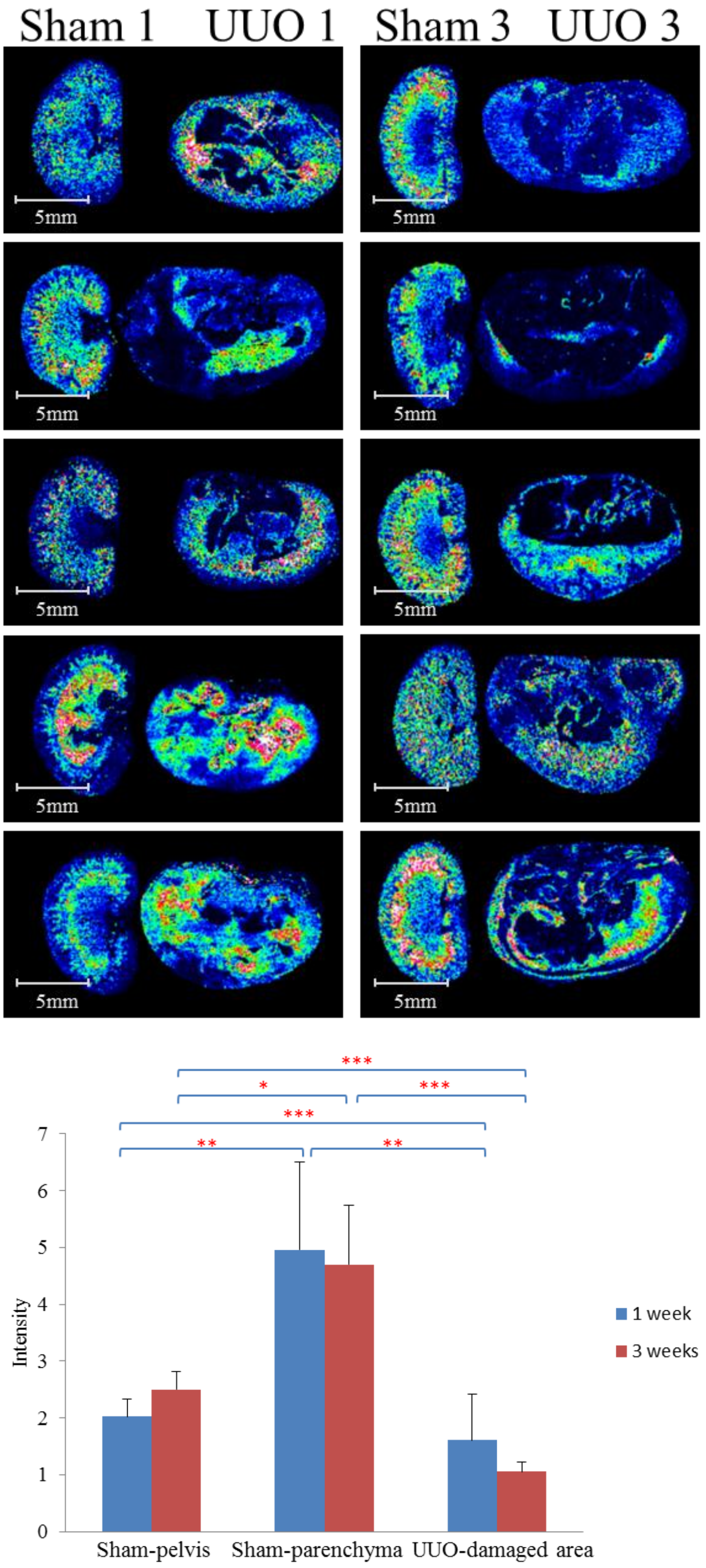

Sham 1
UUO 1
Sham 3
UUO 3
5mm
Intensity
0
1
2
3
4
5
6
7
Sham-pelvis
Sham-parenchyma
UUO-damaged area
1 week
3 weeks
*
**
***

(i) Inosine

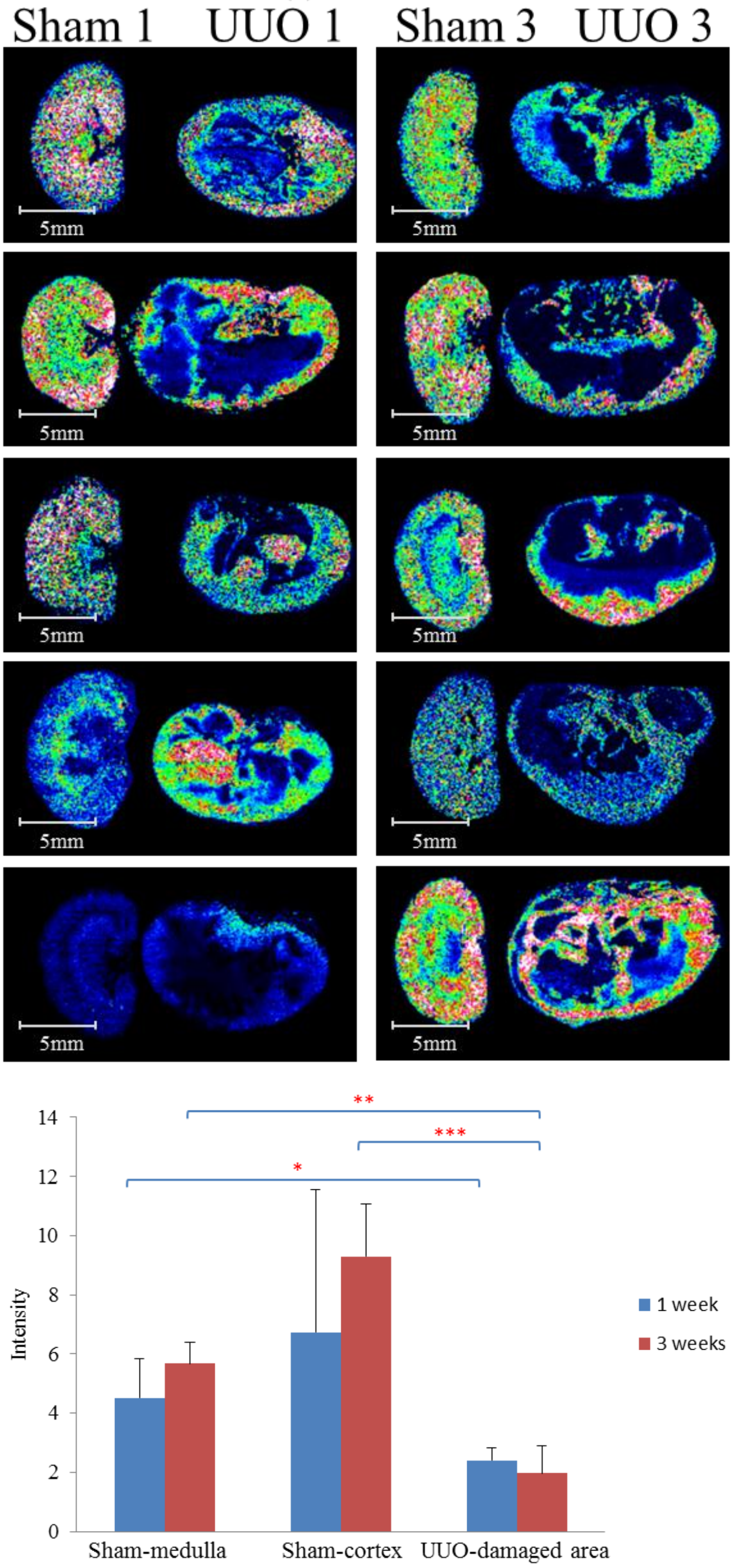

Sham 1
UUO 1
Sham 3
UUO 3
5mm
Intensity
0
2
4
6
8
10
12
14
*
**
***
Sham-medulla
Sham-cortex
UUO-damaged area
1 week
3 weeks

(j) Hypoxanthine

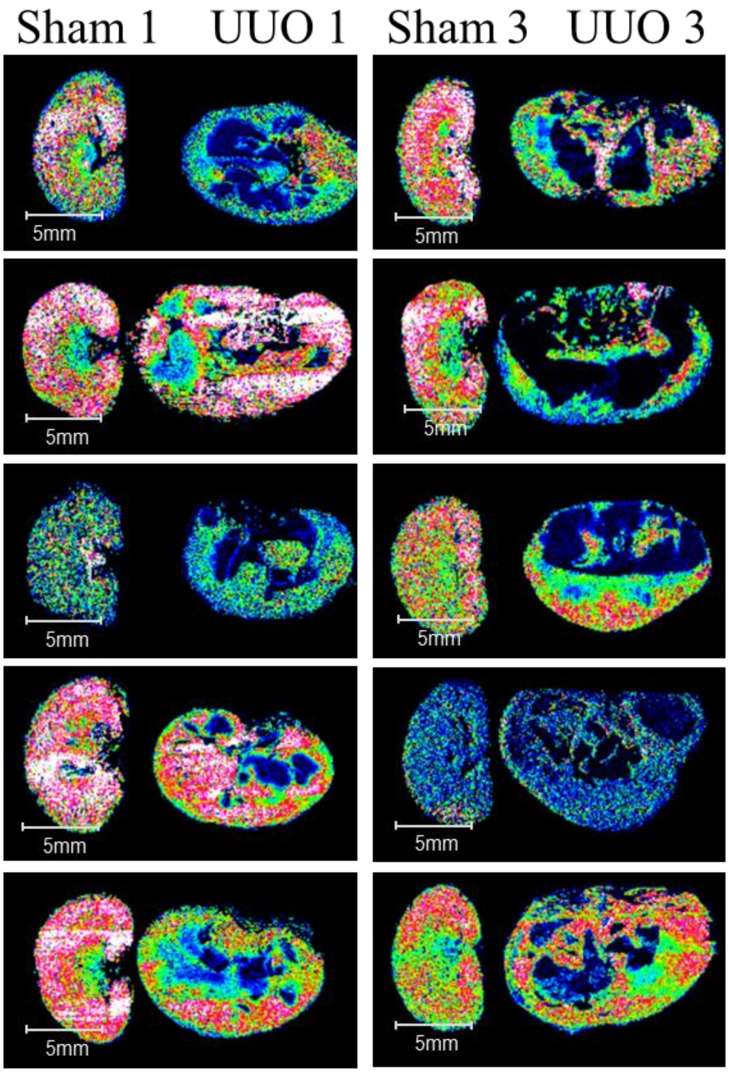

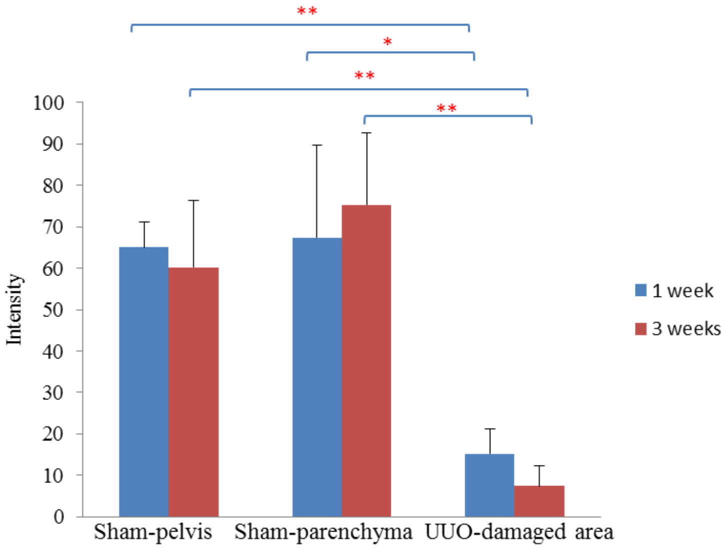

(k) Xanthine

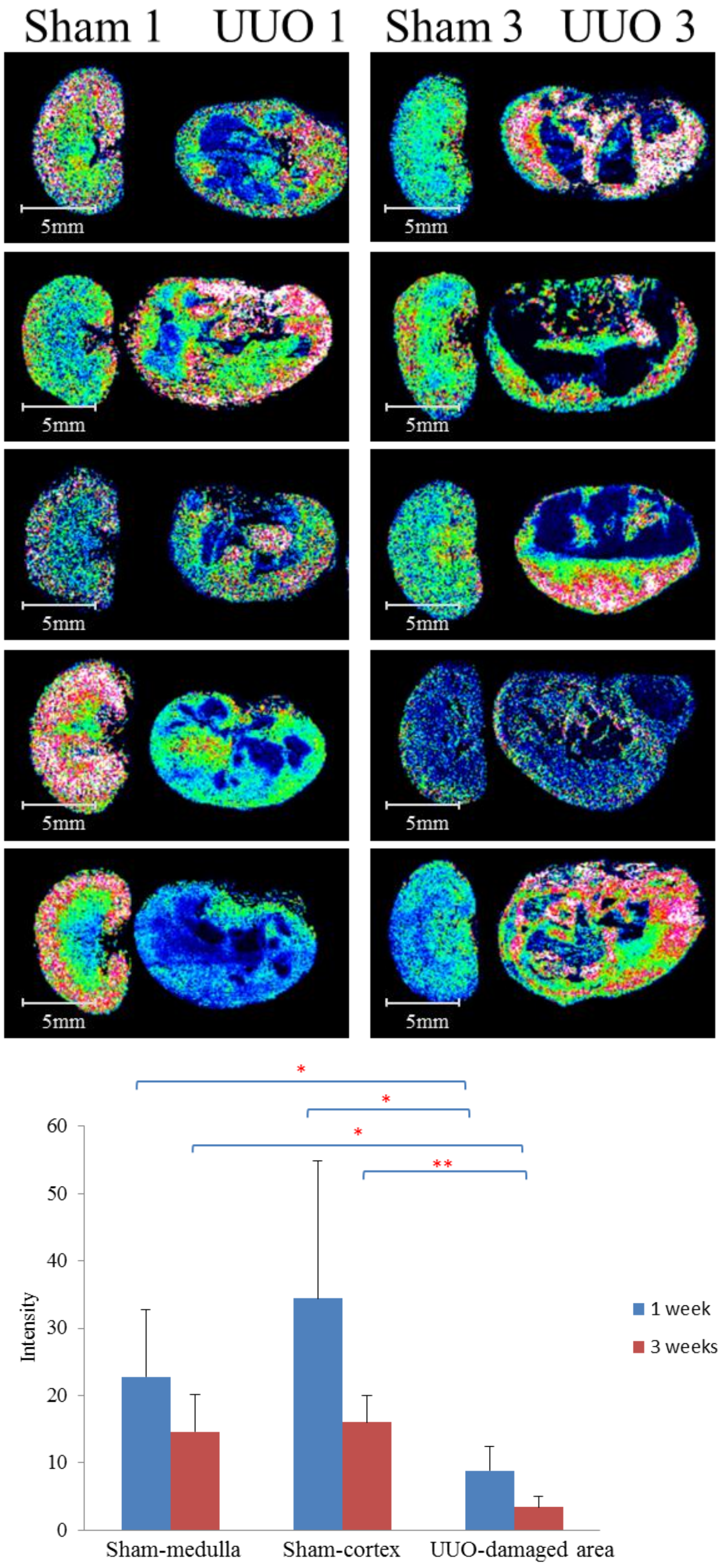
Sham 1
UUO 1
Sham 3
UUO 3
5mm
*
*
*
**
Intensity
0
10
20
30
40
50
60
Sham-medulla
Sham-cortex
UUO-damaged area
1 week
3 weeks

(l) Linoleic acid

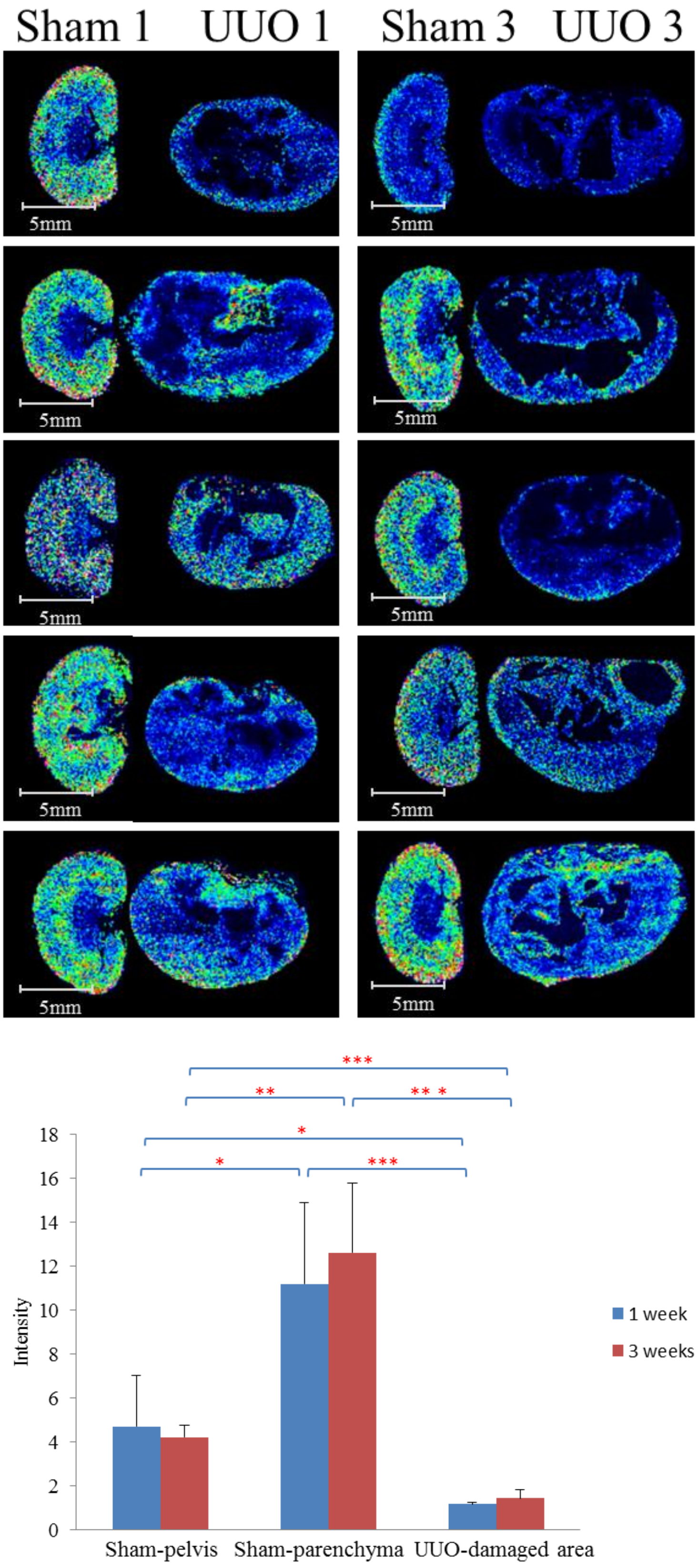

(m) Oleic acid

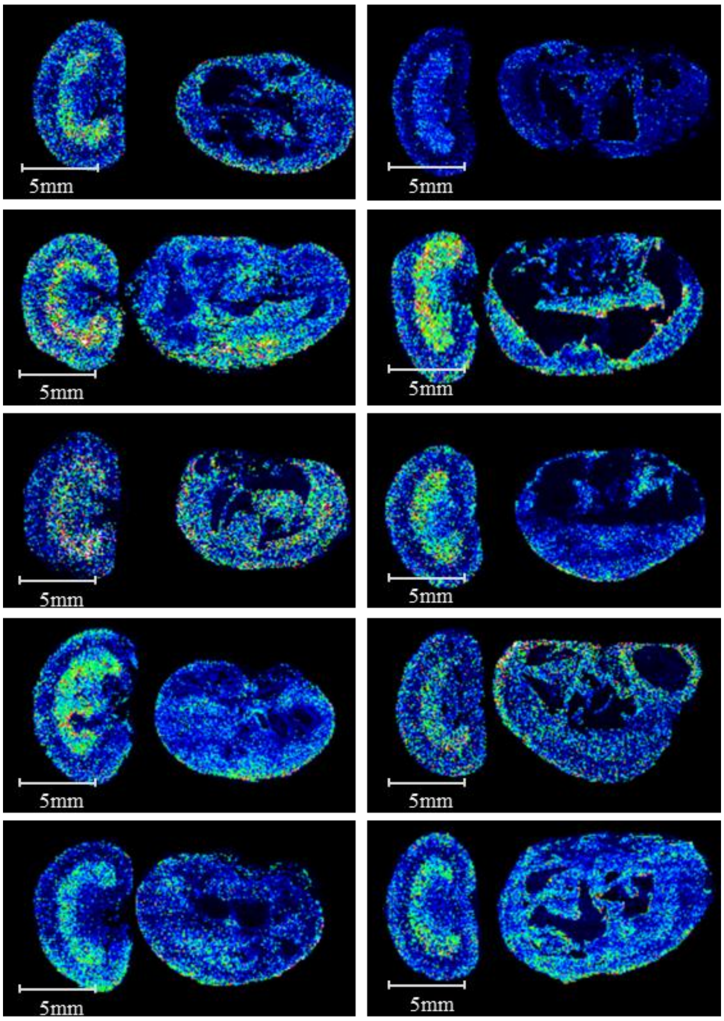
5mm

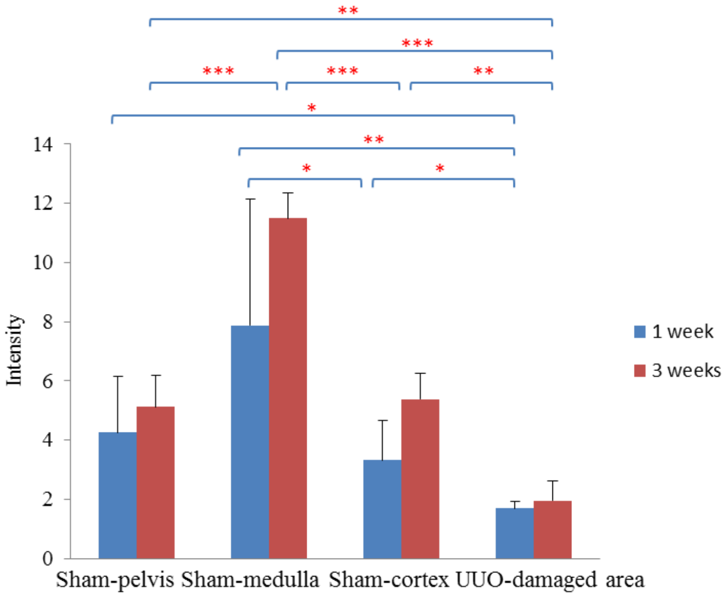
**
***
*** *** **
*
**
* *
Intensity
0
2
4
6
8
10
12
14
Sham-pelvis Sham-medulla Sham-cortex UUO-damaged area
1 week
3 weeks

(n) Stearic acid

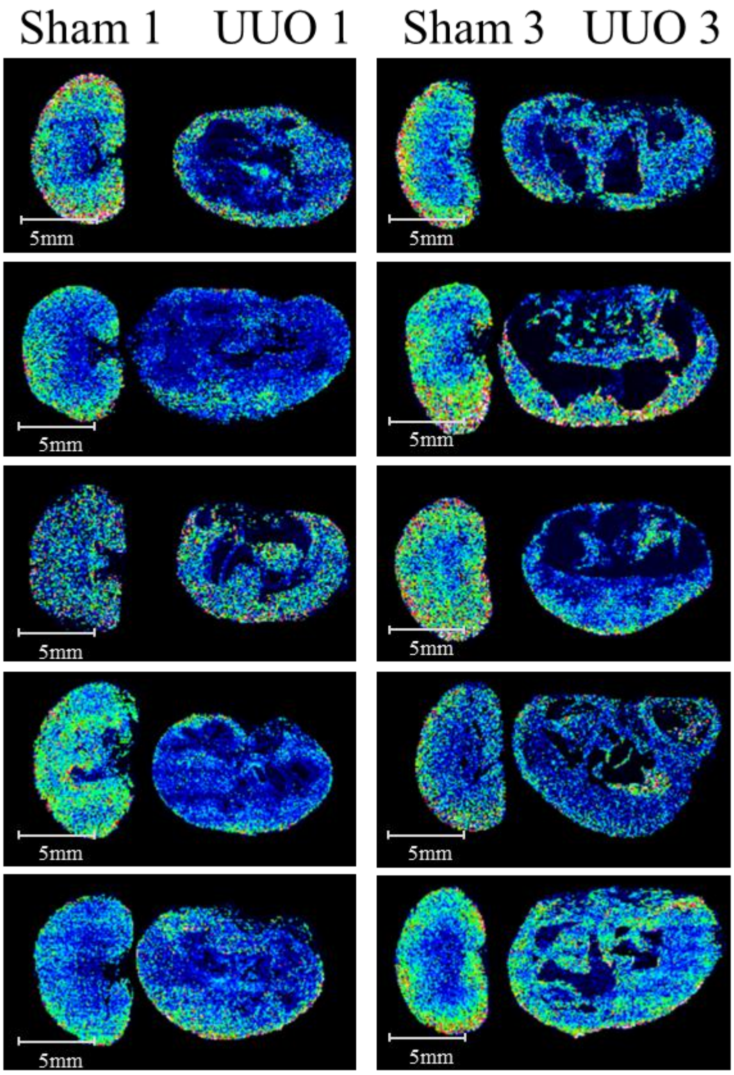

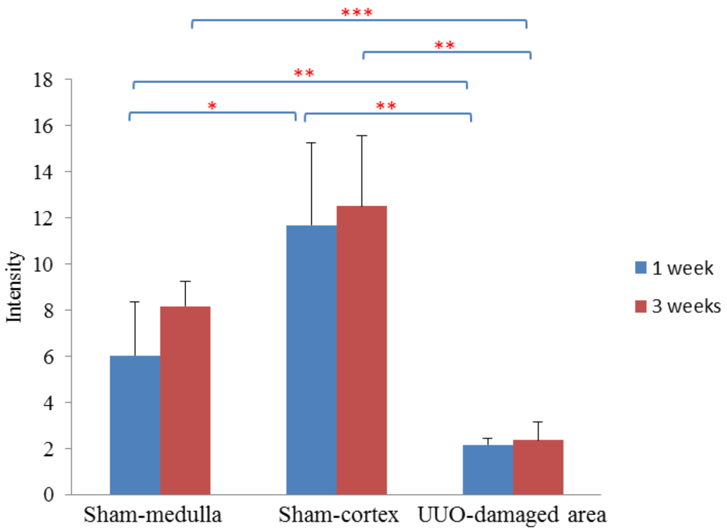

(o) Arachidonic acid

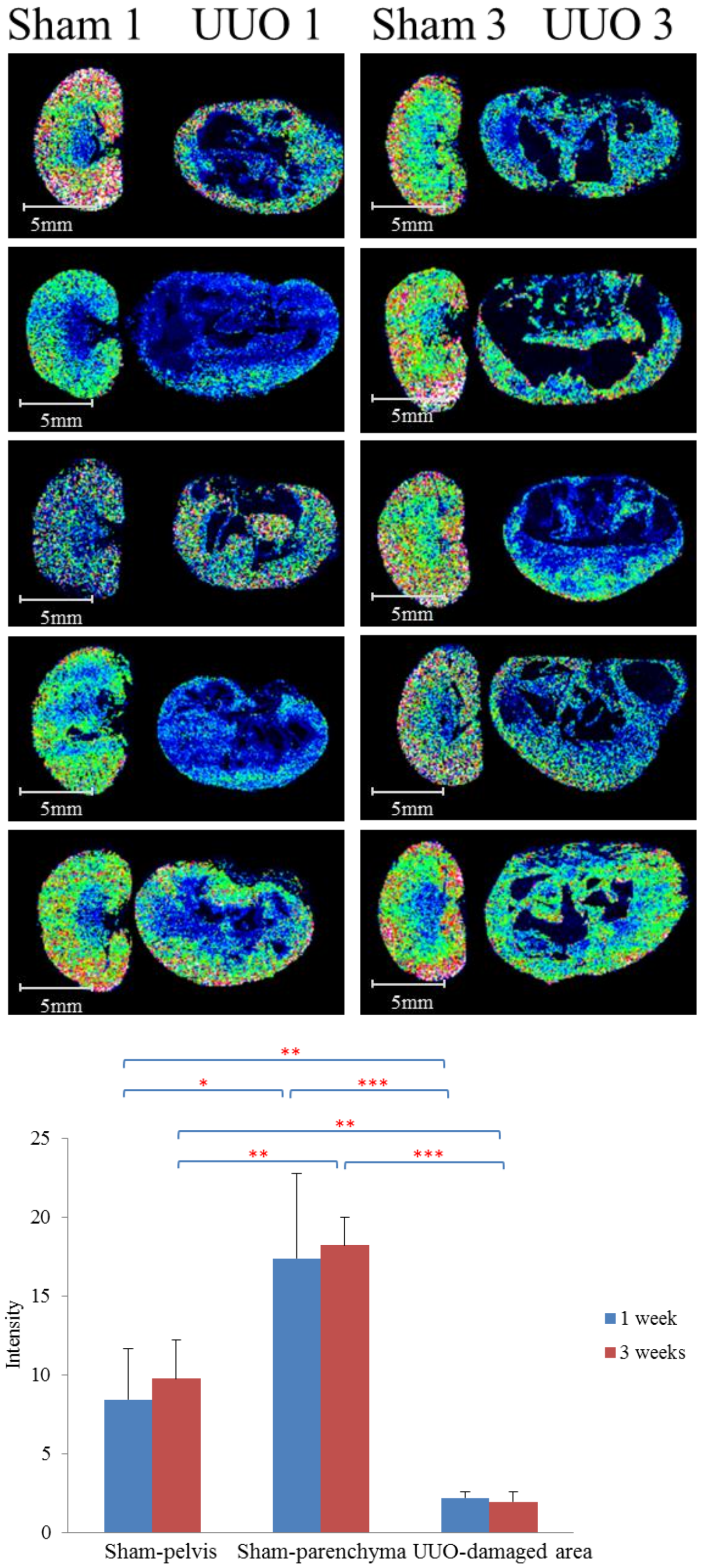

(p) Taurine

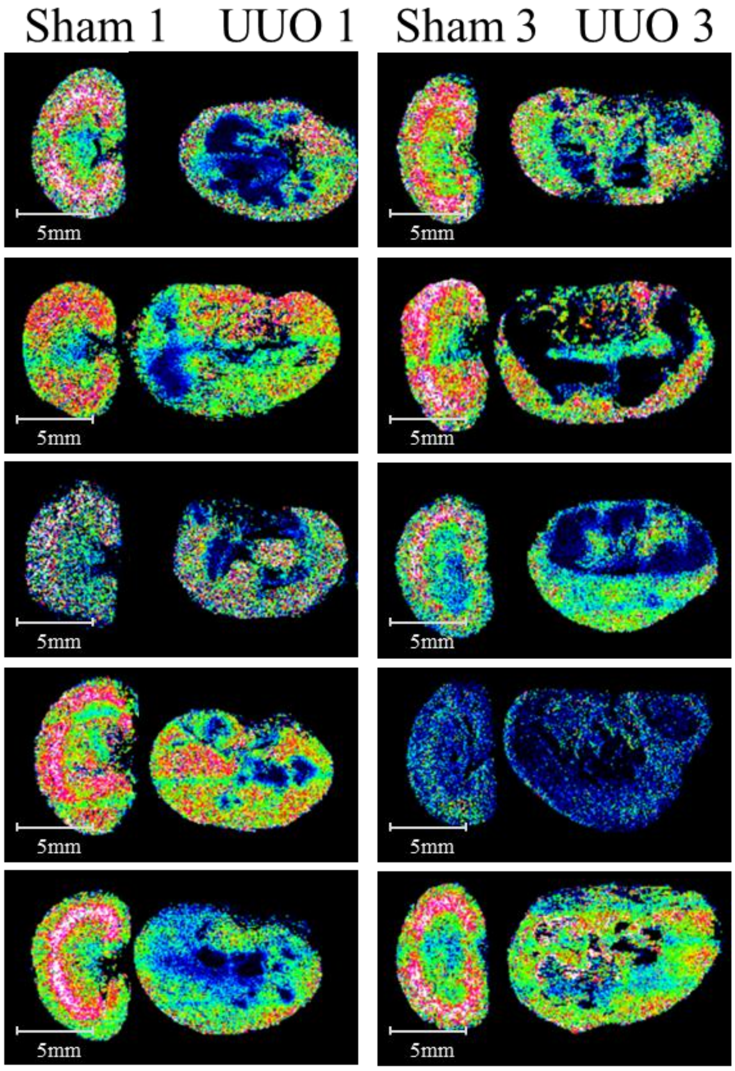
Sham 1
UUO 1
Sham 3
UUO 3
5mm

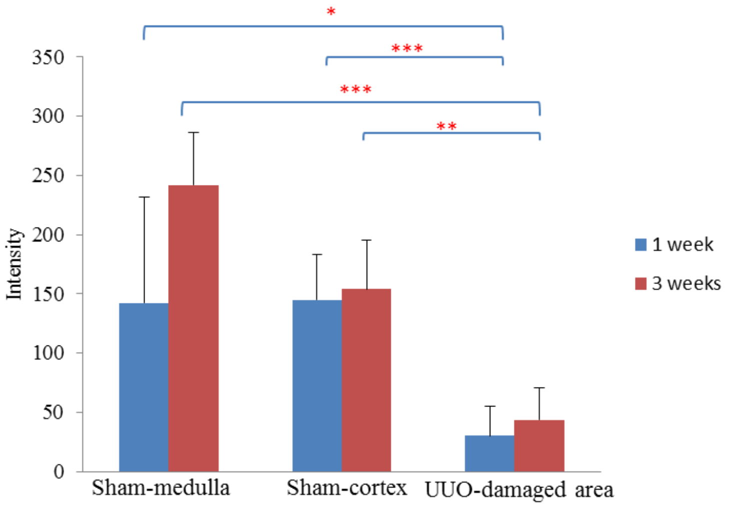
*
***
***
**
350
300
250
200
150
100
50
0
Intensity
Sham-medulla
Sham-cortex
UUO-damaged area
1 week
3 weeks

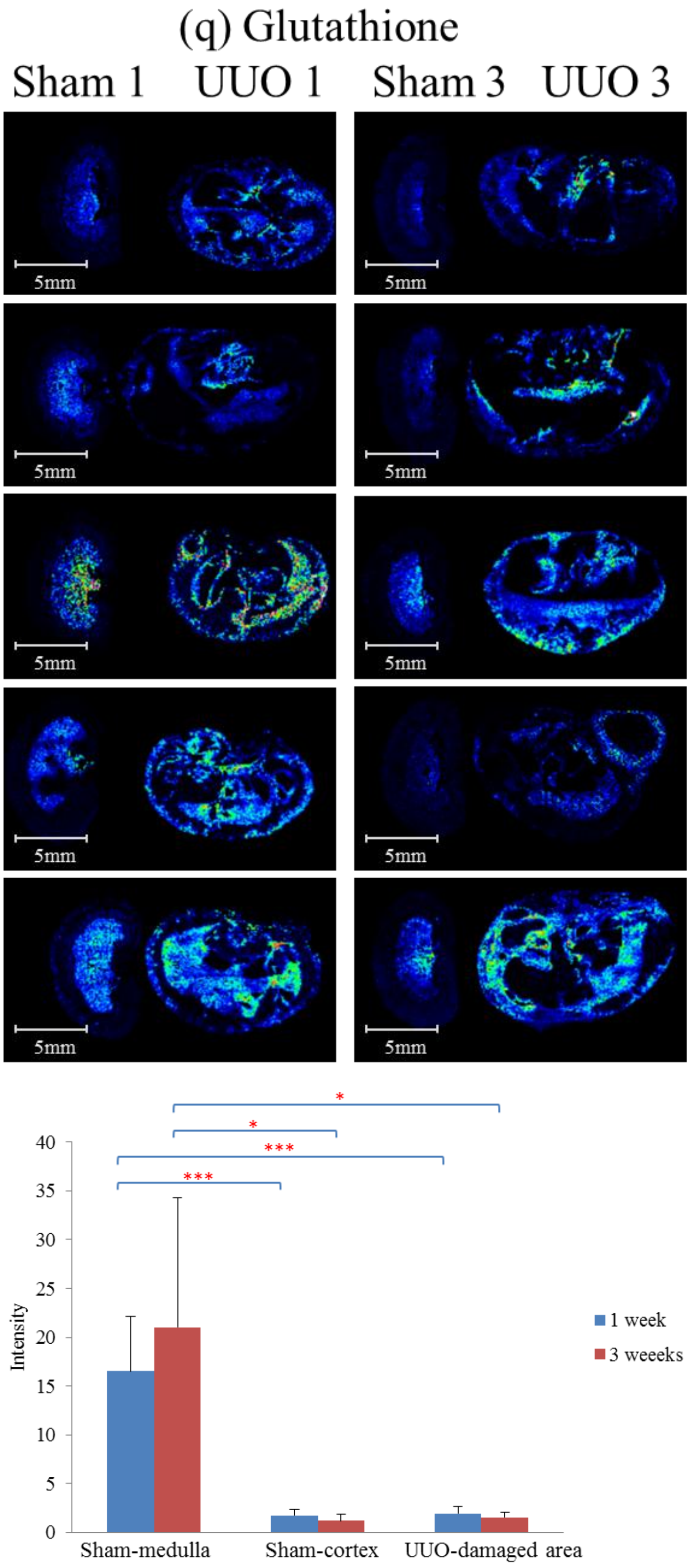
(q) Glutathione
Sham 1
UUO 1
Sham 3
UUO 3
5mm
Intensity
0
5
10
15
20
25
30
35
40
Sham-medulla
Sham-cortex
UUO-damaged area
1 week
3 weeeks
*
*
***
***

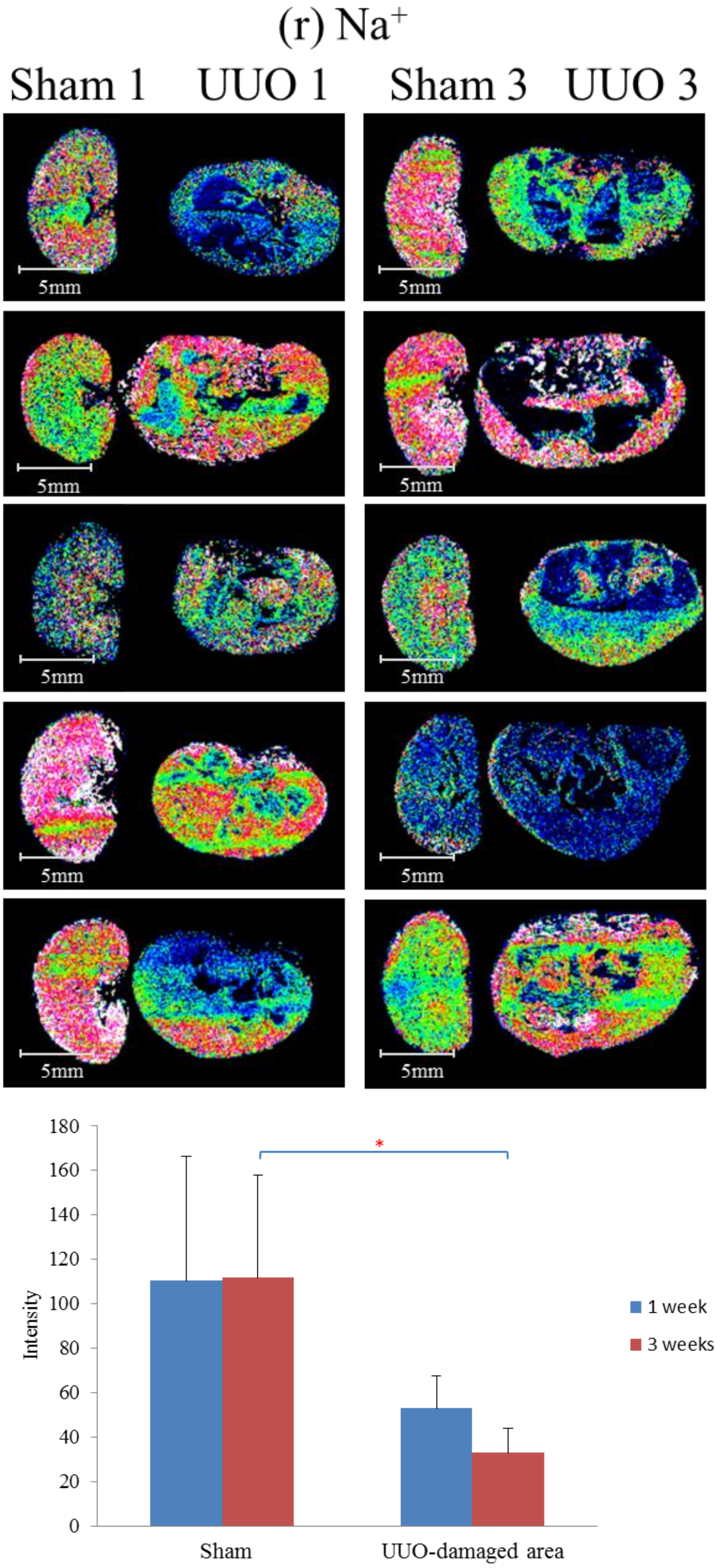
(r) Na^+
Sham 1
UUO 1
Sham 3
UUO 3
5mm
180
160
140
120
100
80
60
40
20
0
Intensity
*
1 week
3 weeks
Sham
UUO-damaged area

(s) K^+

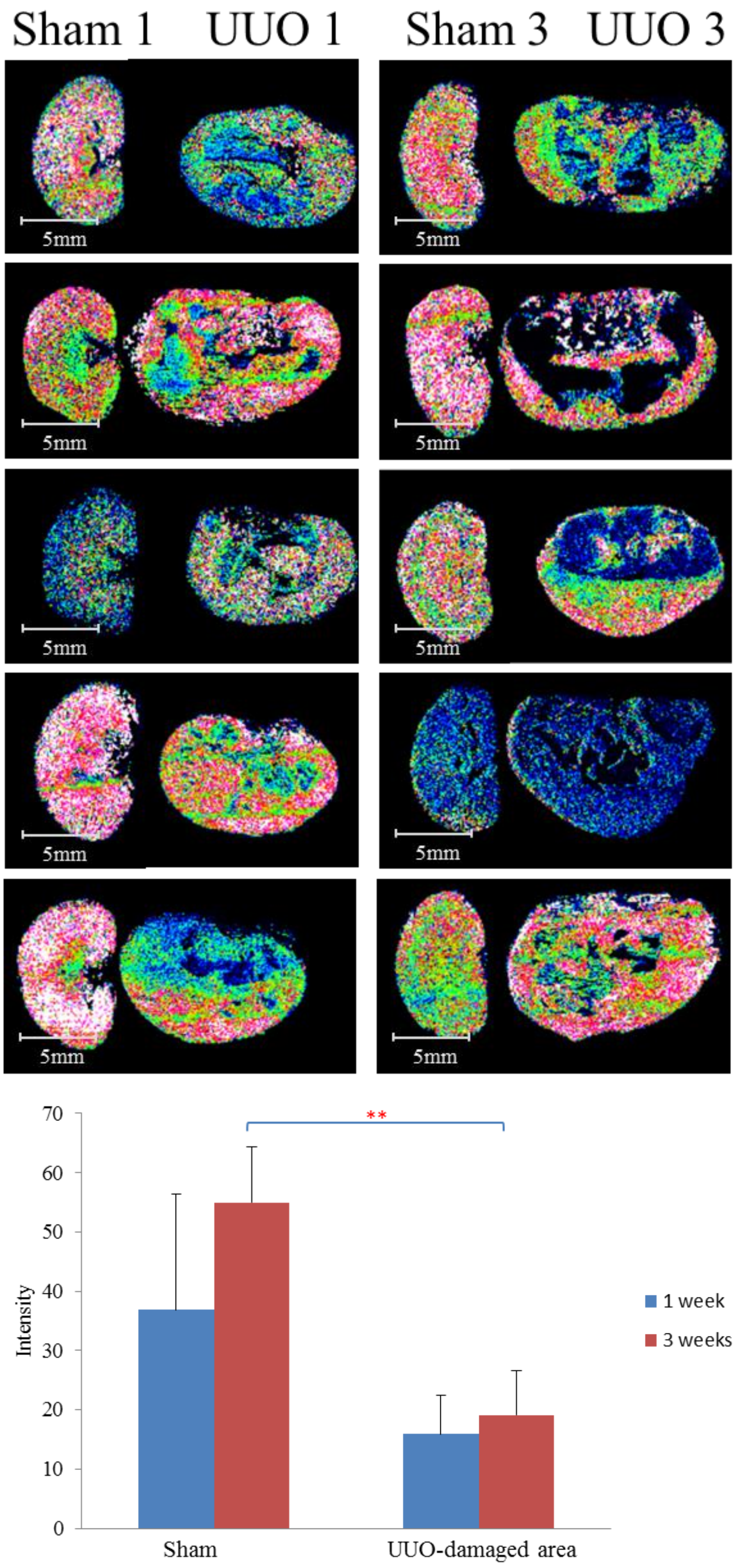
Sham 1
UUO 1
Sham 3
UUO 3
5mm
Intensity
70
60
50
40
30
20
10
0
**
Sham
UUO-damaged area
1 week
3 weeks

(t) Glycerol 3-phosphate

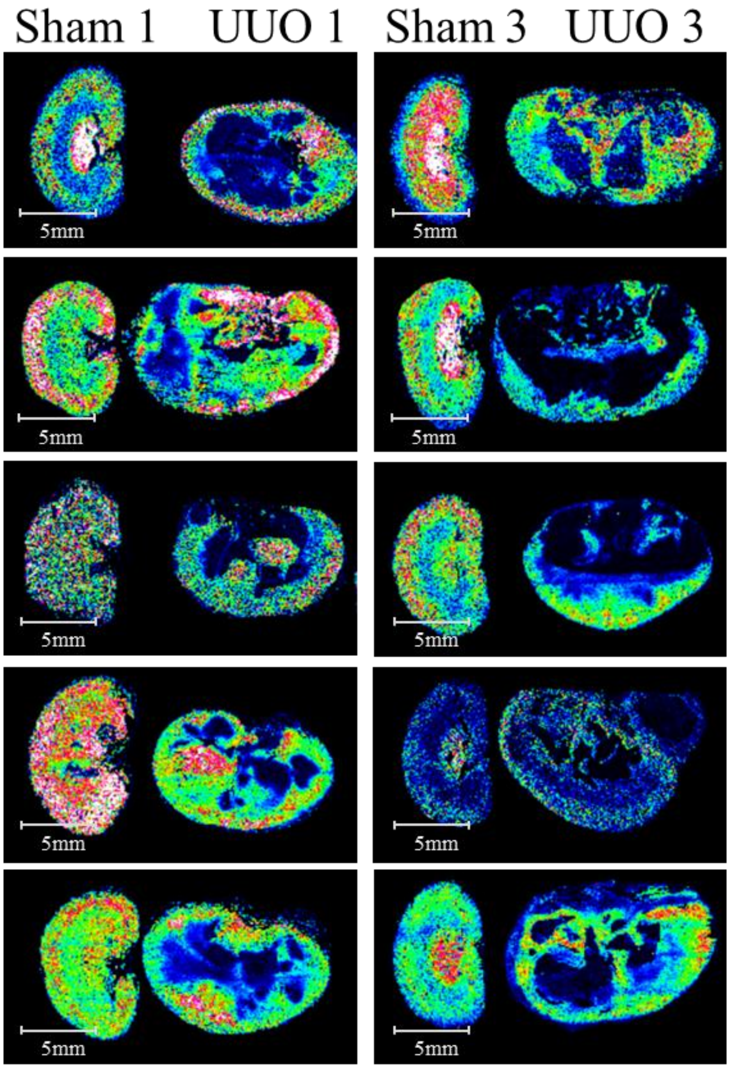

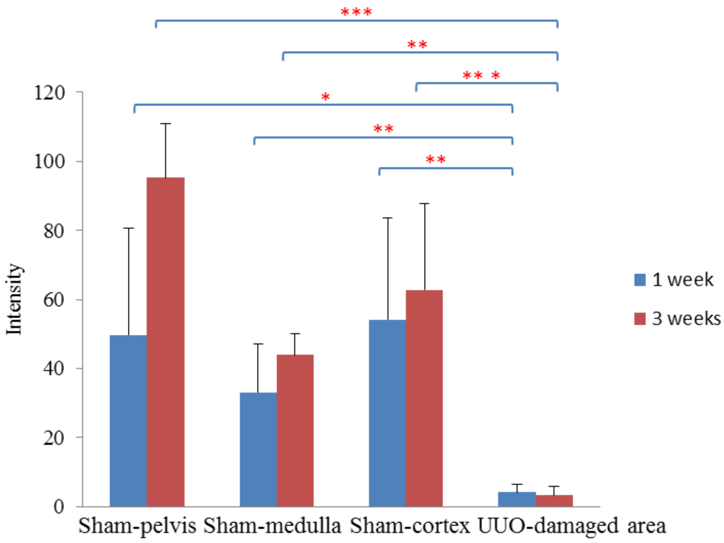

(u) Hippuric acid

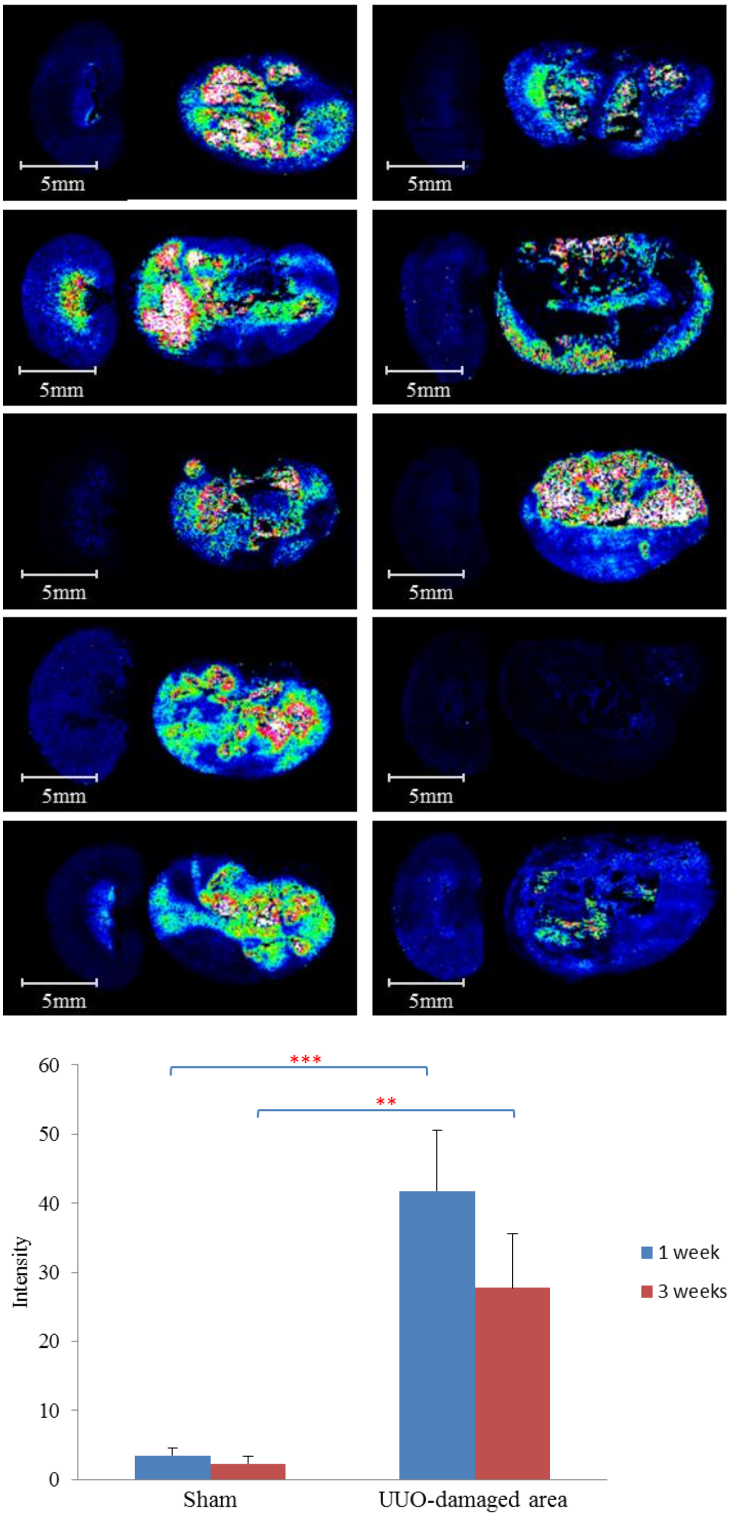

Unconfirmed-1

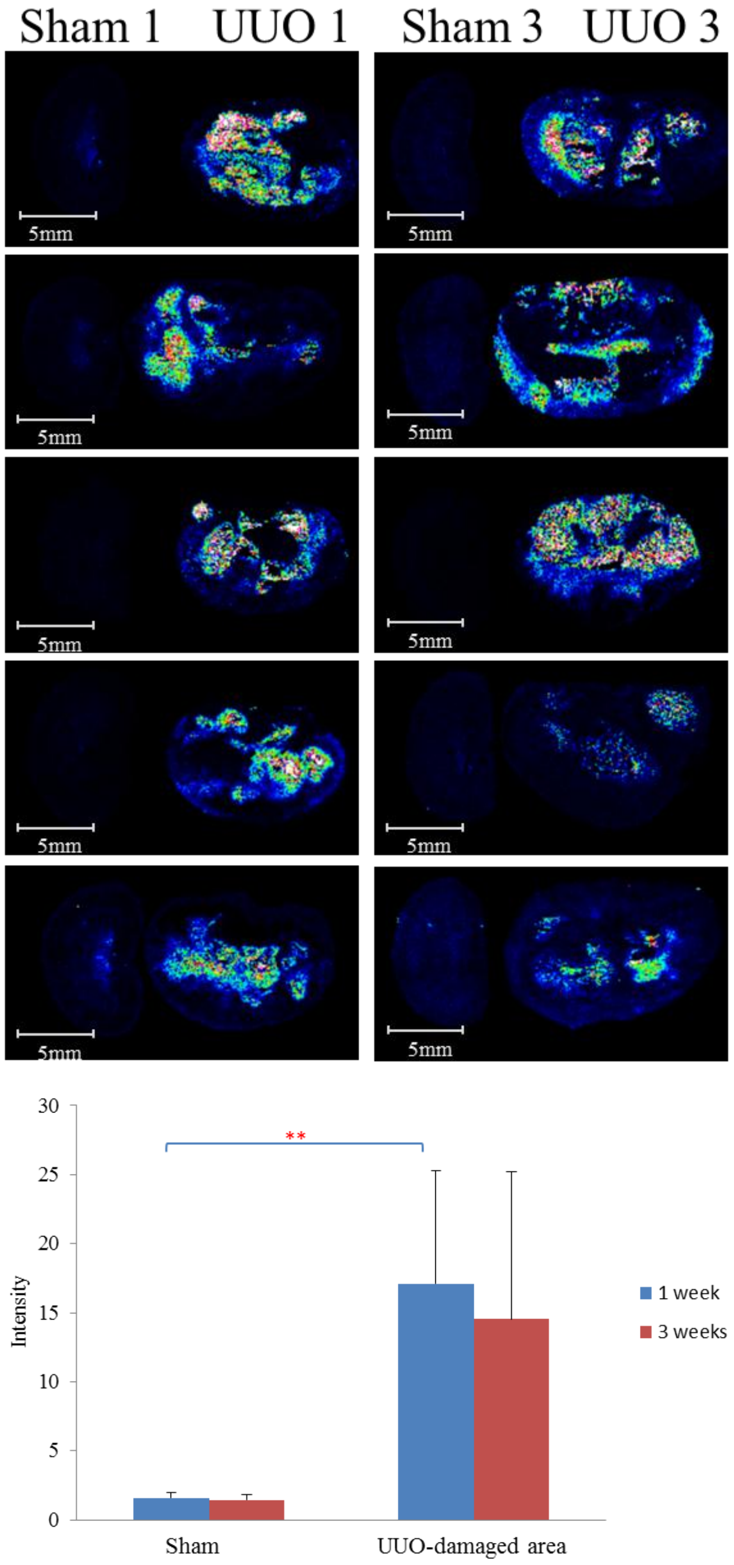

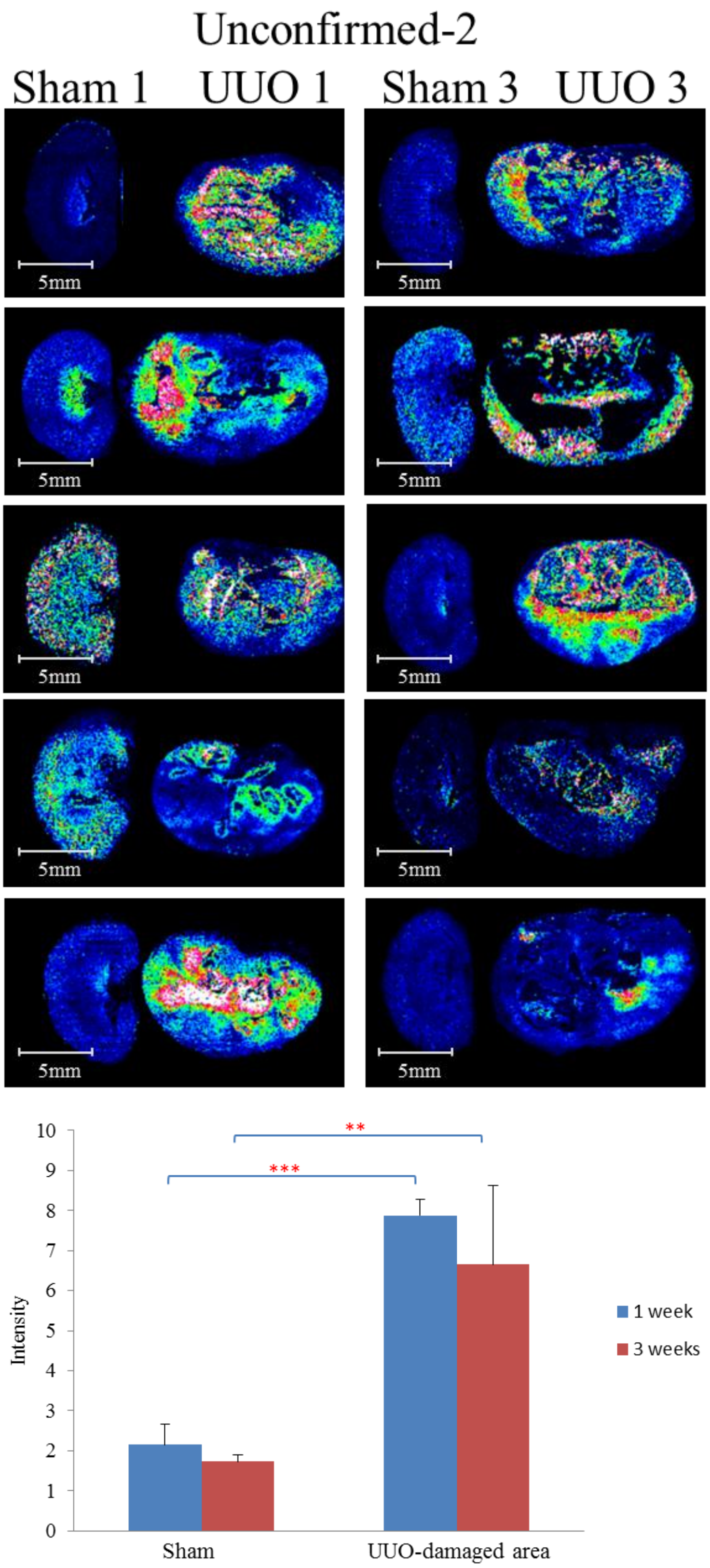
Unconfirmed-2
Sham 1
UUO 1
Sham 3
UUO 3
5mm
Intensity
Sham
UUO-damaged area
1 week
3 weeks
**
***

Unconfirmed-3

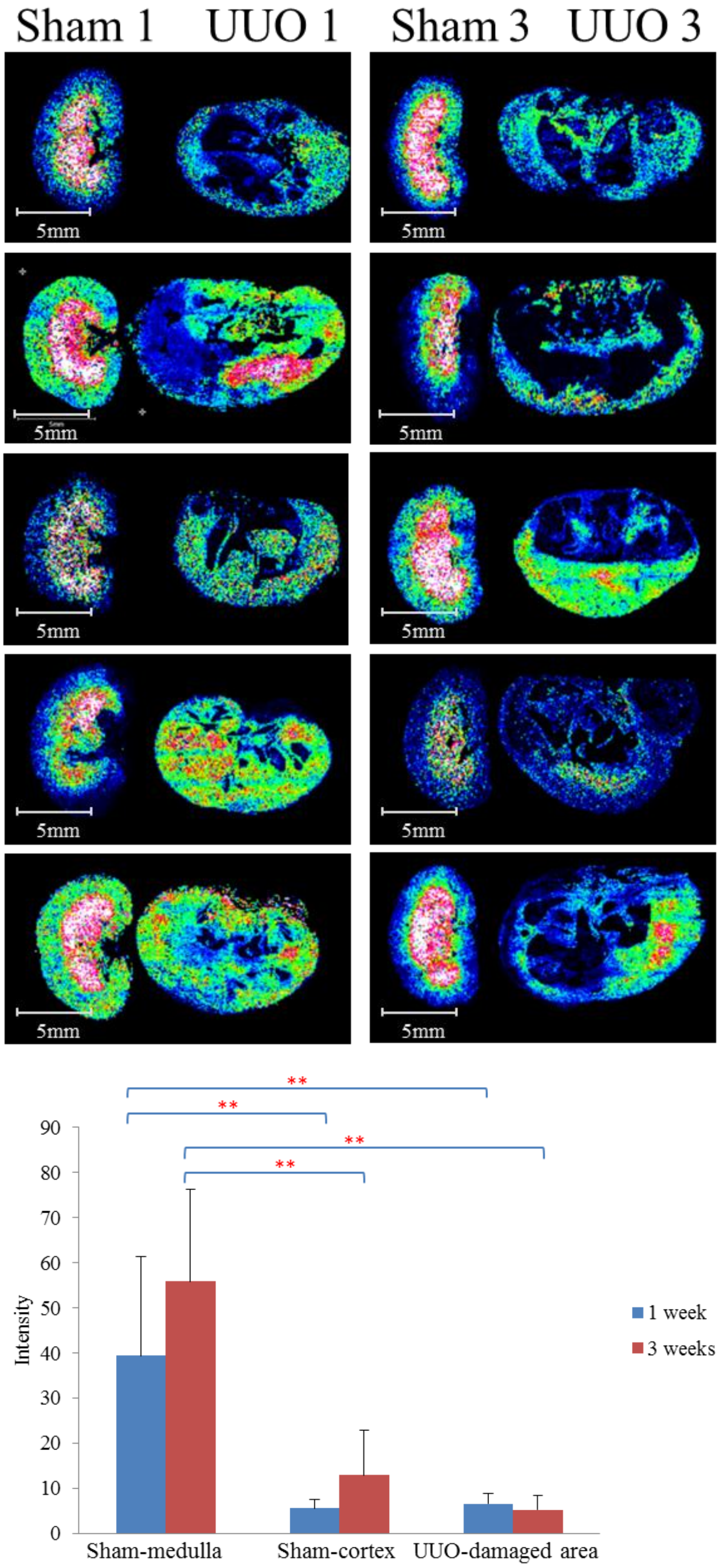

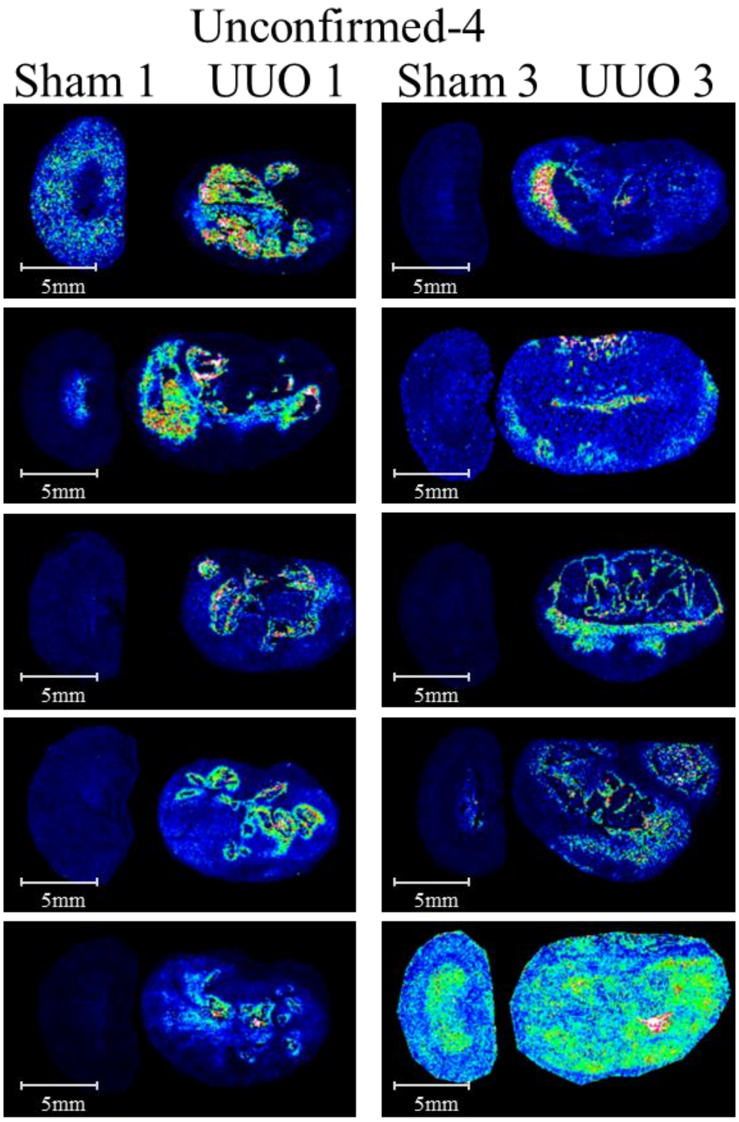
Unconfirmed-4
Sham 1
UUO 1
Sham 3
UUO 3
5mm

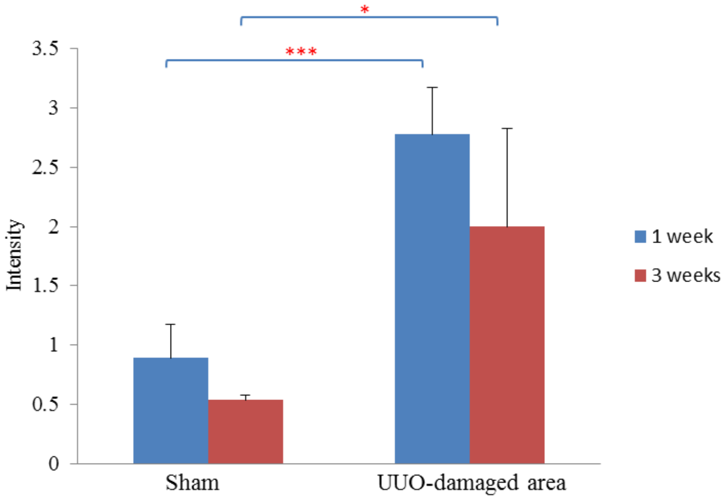
*
***
3.5
3
2.5
2
1.5
1
0.5
0
Intensity
Sham
UUO-damaged area
1 week
3 weeks

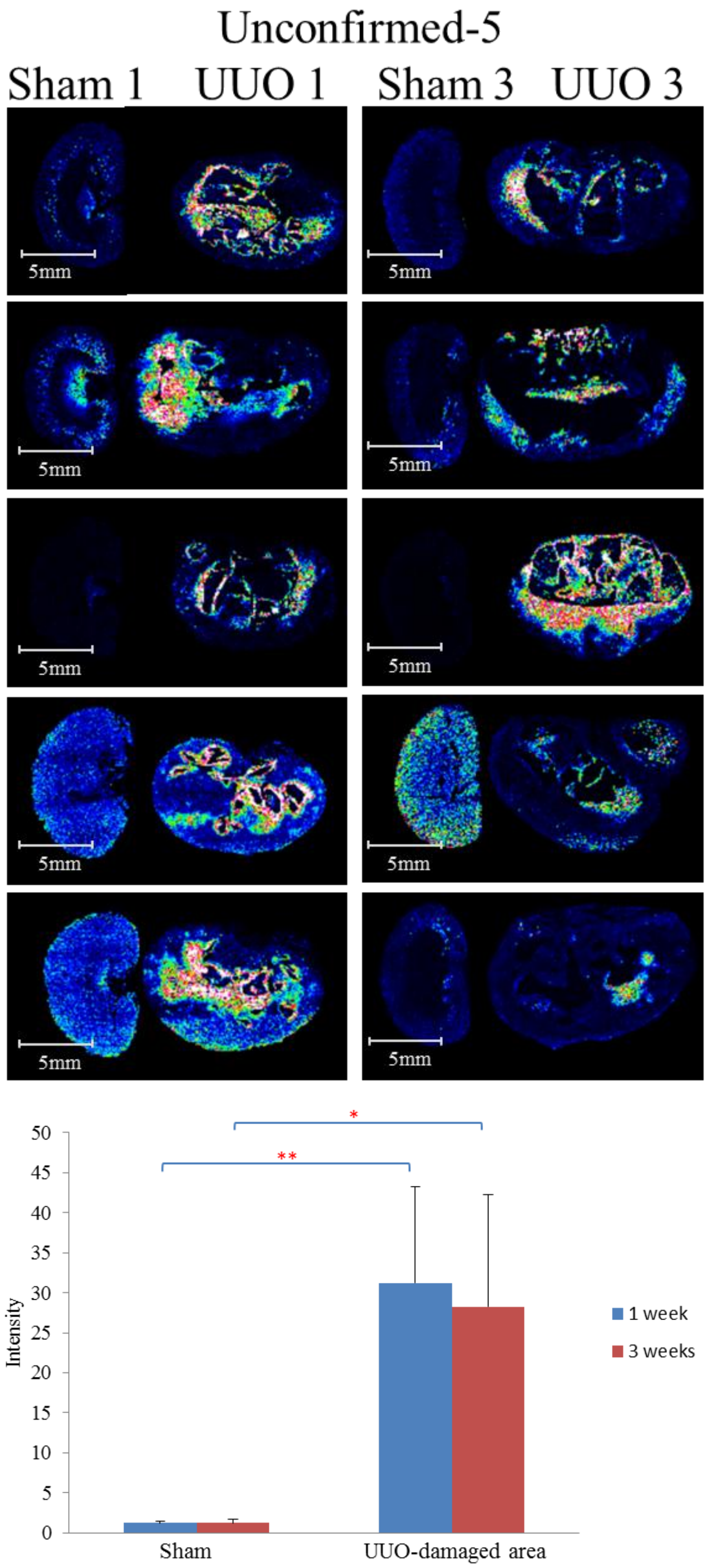
Unconfirmed-5
Sham 1
UUO 1
Sham 3
UUO 3
5mm
50
45
40
35
30
25
20
15
10
5
0
Intensity
*
**
1 week
3 weeks
Sham
UUO-damaged area
